# Diverse RNA-Binding Proteins Interact with Functionally Related Sets of RNAs, Suggesting an Extensive Regulatory System 

**DOI:** 10.1371/journal.pbio.0060255

**Published:** 2008-10-28

**Authors:** Daniel J Hogan, Daniel P Riordan, André P Gerber, Daniel Herschlag, Patrick O Brown

**Affiliations:** 1 Department of Biochemistry, Stanford University School of Medicine, Stanford, California, United States of America; 2 Howard Hughes Medical Institute, Stanford University School of Medicine, Stanford, California, United States of America; 3 Department of Genetics, Stanford University School of Medicine, Stanford, California, United States of America; 4 Institute of Pharmaceutical Sciences, Department of Chemistry and Applied Biosciences, ETH Zurich, Zurich, Switzerland

## Abstract

RNA-binding proteins (RBPs) have roles in the regulation of many post-transcriptional steps in gene expression, but relatively few RBPs have been systematically studied. We searched for the RNA targets of 40 proteins in the yeast Saccharomyces cerevisiae: a selective sample of the approximately 600 annotated and predicted RBPs, as well as several proteins not annotated as RBPs. At least 33 of these 40 proteins, including three of the four proteins that were not previously known or predicted to be RBPs, were reproducibly associated with specific sets of a few to several hundred RNAs. Remarkably, many of the RBPs we studied bound mRNAs whose protein products share identifiable functional or cytotopic features. We identified specific sequences or predicted structures significantly enriched in target mRNAs of 16 RBPs. These potential RNA-recognition elements were diverse in sequence, structure, and location: some were found predominantly in 3′-untranslated regions, others in 5′-untranslated regions, some in coding sequences, and many in two or more of these features. Although this study only examined a small fraction of the universe of yeast RBPs, 70% of the mRNA transcriptome had significant associations with at least one of these RBPs, and on average, each distinct yeast mRNA interacted with three of the RBPs, suggesting the potential for a rich, multidimensional network of regulation. These results strongly suggest that combinatorial binding of RBPs to specific recognition elements in mRNAs is a pervasive mechanism for multi-dimensional regulation of their post-transcriptional fate.

## Author Summary

Regulation of gene transcription has been extensively studied, but much less is known about how the fates of the resulting mRNA transcripts are regulated. We were intrigued by the fact that while most eukaryotic genomes encode hundreds of RNA-binding proteins (RBPs), the targets and regulatory roles of only a small fraction of these proteins have been characterized. In this study, we systematically identified the RNAs associated with a select sample of 40 of the approximately 600 predicted RBPs in the budding yeast, Saccharomyces cerevisiae. We found that most of these RBPs bound specific sets of mRNAs whose protein products share physiological themes or similar locations within the cell. For 16 of the 40 RBPs, we identified sequence motifs significantly enriched in their RNA targets that presumably mediate recognition of the target by the RBP. The intricate, overlapping patterns of mRNAs associated with RBPs suggest an extensive combinatorial system for post-transcriptional regulation, involving dozens or even hundreds of RBPs. The organization and molecular mechanisms involved in this regulatory system, including how RBP–mRNA interactions are integrated with signal transduction systems and how they affect the fates of their RNA targets, provide abundant opportunities for investigation and discovery.

## Abstract 3

A systematic study of the RNA targets of 40 known or predicted RNA-binding proteins in yeast suggests that an extensive system of dozens or hundreds of specific RNA-binding proteins may act to regulate the post-transcriptional fate of most or all RNAs in the yeast cell.

## Introduction

Much of the regulation of eukaryotic gene expression programs is still unaccounted for. Although these programs are subject to regulation at many steps, most investigation has focused on regulation of transcription. There are clues, however, that a significant portion of undiscovered regulation might be post-transcriptional, acting to regulate mRNA processing, localization, translation, and decay [1–5]. For example, systematic phylogenetic comparison among yeast and mammalian genomes sequences have revealed that untranslated regions of many mRNAs are under purifying selection, and thus presumably carrying information important for fitness [6–8].

Biological regulation can be achieved by controlling any of a large number of steps in the lives of RNA molecules. Alternative splicing of transcripts can enable a single gene to encode numerous protein products, greatly expanding its molecular complexity [9]. Even in organisms with few introns, such as *Saccharomyces cerevisiae*, splicing is subject to regulation [10,11]. Notable examples of regulated RNA localization include mRNA export from the nucleus to the cytoplasm, partitioning of mRNAs to the rough endoplasmic reticulum (ER) membrane for cotranslational export, and the precise subcellular localization of thousands of specific mRNAs [12]. In a recent survey of mRNA localization in developing *Drosophila* embryos, more than 70% of the roughly 3,000 mRNAs examined showed distinct patterns of subcellular localization [13]. Widespread regulation of translation rates is evident in several observations. In yeast, despite extensive regulation of transcription and mRNA decay, only about 70% of the observed variance in protein abundance is accounted for by variation in mRNA abundance [14,15]. When cells are moved from rich media to minimal media, the abundance of hundreds of proteins change, but mRNA abundance changes parallel changes in the abundance for only about half of the cognate proteins [16,17]. The abundance of each RNA is determined jointly by regulated transcription and regulated degradation. Widespread, transcript-specific regulation of mRNA decay is evident from the closely matched decay rates of mRNAs encoding functionally related proteins [18–21], particularly evident in S. cerevisiae in sets of proteins that form stoichiometric complexes [19].

Increasing evidence points to extensive involvement of specific RNA-binding proteins (RBPs) in regulation of these post-transcriptional events [1–5]. Pioneering studies focusing on tens of predominantly nuclear mRNA RBPs (so-called heterogeneous ribonucleoprotein [hnRNP] proteins), revealed that these proteins recognize specific features in mRNAs, bind at overlapping, but distinct, times during RNA processing, and differentially associate with subsets of nascent transcripts [22]. Steps in RNA processing in the nucleus are functionally and physically coupled, providing an opportunity for coordinated control [23].

Investigations of regulation acting on RNA have usually focused on a few model RNAs, leaving unanswered the extent to which mRNAs are coordinated and differentially regulated, and this regulatory landscape is still largely unexplored. Recent studies have systematically identified the suite of mRNAs associated with some individual RBPs. Several RBPs implicated in RNA processing and nuclear export in S. cerevisiae were found to associate with distinct sets of hundreds of functionally related mRNAs [24,25]. Five members of the Puf family of RBPs in S. cerevisiae were each found to associate with distinct, overlapping sets of 40–250 mRNAs [26]. The specific sets of mRNAs associated with each Puf protein were significantly enriched for mRNAs encoding functionally and cytotopically related proteins. For instance, most of the approximately 220 mRNAs associated with Puf3 are transcribed from nuclear genes and encode proteins localized to the mitochondrion (*p* < 10^−100^). Puf3, Puf4, and Puf5 each recognize specific sequences in the 3′-untranslated regions (UTRs) of their targets. These results and others, from studies of a few selected RBPs, may be just a glimpse of a much larger and richer post-transcriptional regulatory network, involving dozens to hundreds of RBPs and a cognate suite of recognition elements in their RNA targets (e.g., [22,24–40]).

But does such a multidimensional post-transcriptional regulatory network exist? To test this hypothesis and to extend and deepen our understanding of RBP–RNA interactions, we systematically searched for the RNA targets of a select sample of 40 out of the more than 500 known and predicted RBPs in S. cerevisiae.

## Results

### Systematic Identification of RNAs Associated with a Select Sample of RNA-Binding Proteins

We first developed a list of candidate RBPs based on annotations in the *Saccharomyces* Genome Database (SGD) (http://www.yeastgenome.org), the Yeast Protein Database [41], and the Munich Information Center for Protein Sequences database [42] and on literature searches. From the assembled list of 561 genes (Table S1), we chose a set of 36 with diverse RNA-binding domains and diverse functional annotations (Table S2 and Text S1). Because many known RBPs lack recognizable RNA-binding domains, we also included two metabolic enzymes whose homologs in other species are known to associate with RNA, and two proteins that were not, a priori, expected to bind RNA, but which we suspected might have post-transcriptional regulatory functions (Table S2).

To identify RNAs associated with each putative RBP, C-terminal tandem affinity purification (TAP)-tagged proteins, expressed under control of their native promoters, were affinity purified from whole-cell extracts of cultures grown to mid-log phase in rich medium [14,26,43]. Extracts were incubated with immunoglobulin G (IgG) agarose beads, washed, and ribonuclear protein complexes were eluted by tobacco etch virus (TEV) protease treatment (Text S2). We performed two to four independent isolations with each tagged strain. As controls, we performed 13 immunoaffinity purifications (IPs) of untagged strains to identify and exclude potential false-positive RNA targets.

We purified total RNA from the whole-cell extracts and TEV-purified fractions, reverse transcribed with an amino-allyl-dUTP/dNTP mix, coupled the purified cDNA to Cy3 and Cy5 dyes, respectively, mixed the two differentially labeled cDNA pools, and then hybridized them to DNA microarrays (Dataset S1).

We identified RNAs specifically associated with each protein using the significance analysis of microarrays (SAM) algorithm [44]. Although it is not possible to perfectly distinguish targets from nontargets, and the best criterion for distinguishing targets from nontargets is unlikely to be the same for all proteins, for most proteins, we chose a 1% false discovery rate (FDR) as a criterion for identifying targets (Datasets S2 and S3). For many RBPs, the number of RNAs called significantly enriched has an inflection point near 1% FDR, suggesting that this threshold is a good balance between sensitivity and specificity, but undoubtedly our identification of specific RBP targets is not comprehensive. For two proteins in the survey (Ssd1 and Khd1), we used a more stringent 1% local FDR criterion [45] (details in Materials and Methods; Datasets S2 and S3). We also included mRNAs specifically associated with Puf1–5 from our previous work [26], (defined using a 1% local FDR), and previously identified She2 targets [32].

### Diverse Binding Specificity among RNA-Binding Proteins

**Figure 1:**
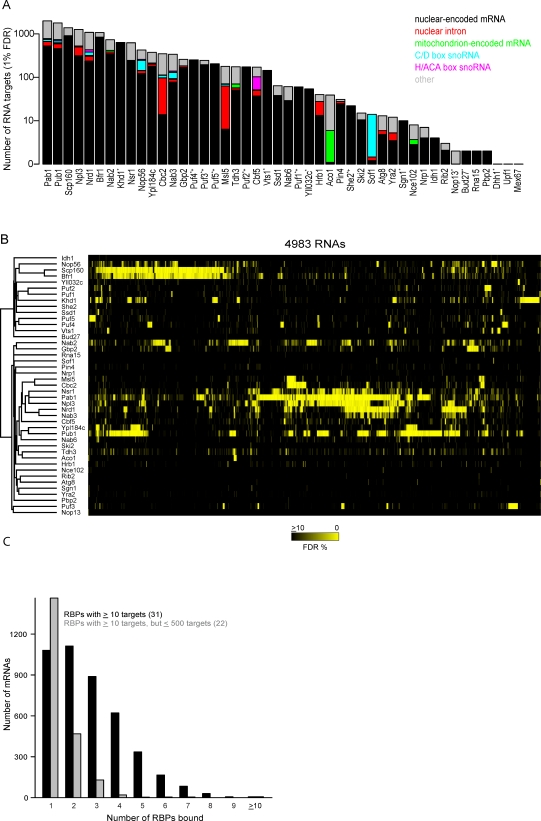
Diverse Binding Specificity among RNA-Binding Proteins (A) Estimated number of RNA species (*y*-axis) associated with each protein in this survey (*x*-axis) at a 1% FDR threshold. The proportions of bound RNAs in each of several classes are represented by colors: nuclear-encoded mRNAs (black), nuclear introns (red), mitochondrion-encoded mRNAs (green), C/D box snoRNAs (cyan), H/ACA box snoRNAs (magenta), and all other RNAs (grey), which includes ribosomal RNAs, LSR1, NME1, SCR1, SRG1, TLC1, mitochondrial introns, unannotated “intergenic” transcripts (named IGR* and IGX* in Datasets S1–S3), tRNAs, and splice junctions. An asterisk (*) denotes proteins whose targets were identified using DNA microarrays that did not contain probes designed to detect most non-nuclear-encoded mRNAs. Six proteins from our previously published work (Puf1–5 and She2) are also included (marked with a plus sign [+]). (B) Hierarchically clustered heat map representation of RBPs and their RNA targets. Rows correspond to specific RBPs and columns correspond to RNAs. The certainty that the RNA is a bona fide target of the specified RBP is represented by a continuous black (10% FDR or greater) to yellow (0% FDR) scale. (C) The distribution of the number of RBPs bound per mRNA at 1% FDR threshold is shown as a bar plot. The black bars represent the values for the 31 RBPs associated with at least ten mRNAs, and the grey bars represent the 22 RBPs associated with at least ten mRNAs, but fewer than 500 mRNAs.

The 40 proteins in the survey (and also Puf1–5 and She2 from our previous work [26,32]) displayed diverse patterns of specificity with regard to the numbers and types of RNA targets and their enrichment profiles (Figures 1 and S1, and Text S3). The number of confidently identified RNA targets varied widely among the proteins surveyed, ranging from fewer than ten (Nce102, Nrp1, Idh1, Rib2, Nop13, Bud27, Rna15, Pbp2, Dhh1, Upf1, and Mex67) to more than a thousand (Pab1, Pub1, Scp160, Npl3, Nrd1, and Bfr1) (Figure 1A). The two “negative controls,” Nce102 and Bud27, were each associated with specific RNAs. Nce102 was associated with eight distinct RNAs, whereas Bud27 was associated with two putative mRNA targets; interestingly, one of these putative targets (RPA190) was reproducibly enriched more than 300-fold, and both targets were lost when immunopurifications were performed in the absence of Mg^2+^ (unpublished data). Because neither Nce102 nor Bud27 was known or expected to associate with RNA, the RNAs identified as their targets may be spurious, but we cannot exclude the possibility that the RNA interactions we found for these two proteins are real and significant. Regardless, they provide a benchmark estimate of the number of RNA targets falsely identified for other RBPs. Aconitase (Aco1) and glyceraldehyde-3 phosphate dehydrogenase (Tdh3), two metabolic enzymes whose human orthologs also function as RBPs [46,47], but which were not previously known to be RBPs in yeast, associated with 38 and 155 RNAs, respectively, at 1% FDR, indicating that these enzymes are also RBPs in yeast.

Fourteen of the proteins we surveyed specifically associated with RNAs other than mature mRNAs encoded by nuclear genes (Figure S2). Their specific targets included intron-containing transcripts (Cbc2, Msl5, Npl3, Hrb1, Pab1, and Pub1), H/ACA box small nucleolar RNAs (snoRNAs) (Cbf5, Nrd1, and Pub1), C/D box snoRNAs (Nop56, Sof1, Nab3, Nrd1, Pub1, and Pab1), and mitochondrial mRNAs (Aco1, Tdh3, and Nab2). Several of these proteins have previously been shown to be associated with specific classes of RNA (Cbc2, Msl5, Npl3, Cbf5, Nrd1, Nop56, Sof1, and Nab3), and therefore provide de facto positive controls (Table S2 and Text S4). Aco1, a TCA cycle enzyme [48], which has recently been implicated in maintaining mitochondrial genome integrity [49], selectively binds transcripts encoded by the mitochondrial genome (*p* < 10^−38^). Our results also suggest unexpected associations for several noncoding-RNA–binding proteins and suggest possible regulatory links between mRNA and noncoding RNA (ncRNA) processing (Text S4). However, the remainder of this report will focus mostly on mRNA targets.

### Most mRNAs Associate with Multiple RNA-Binding Proteins

To explore the interrelationships among RBPs and their RNA targets, we organized RNAs (Figure 1B, columns) and RBPs (Figure 1B, rows), respectively, by hierarchical clustering based on their patterns of mutual interactions, and visualized the results as a heat map representing the confidence of an RNA–RBP interaction with a black (>10% FDR) to yellow (0% FDR) scale. For the most part, each RBP had a unique profile of enrichment, with a few notable exceptions, including Scp160/Bfr1 and Nrd1/Nab3, which are pairs of proteins that act together in stable stoichiometric complexes [50,51] and were correspondingly associated with similar sets of mRNAs.

Altogether, we identified more than 12,000 mRNA–RBP interactions (at a 1% FDR), an average of at least 2.8 RBPs interacting with each of 4,300 distinct mRNAs; 31 proteins (including Puf1–5 and She2) reproducibly bound at least ten mRNAs (at a 1% FDR). Most mRNAs were bound by multiple RBPs (Figure 1C, black bars); 628 mRNAs were bound by five or more of this set of 31 RBPs; intriguingly, a disproportionate fraction of the mRNAs with the greatest number of identified interactions with this set of RBPs encode proteins localized to the cell wall (31, *p* < 10^−4^).

About 75% (∼9,000) of the mRNA–RBP interactions identified in this survey were accounted for by the nine proteins that targeted more than 500 mRNAs each, (Figure 1C, grey bars). Our conservative approach to target identification, emphasizing specificity over sensitivity, probably underestimates the number of targets of these broad-specificity RBPs; some of these proteins, such as Scp160 and Pab1, probably bind most or all mRNAs (Figure S1 and Text S3). The specificity and regulatory contributions of these “general” RBPs are still poorly understood.

### Many RNA-Binding Proteins Associate with mRNAs Encoding Functionally and Cytotopically Related Proteins

**Figure 2:**
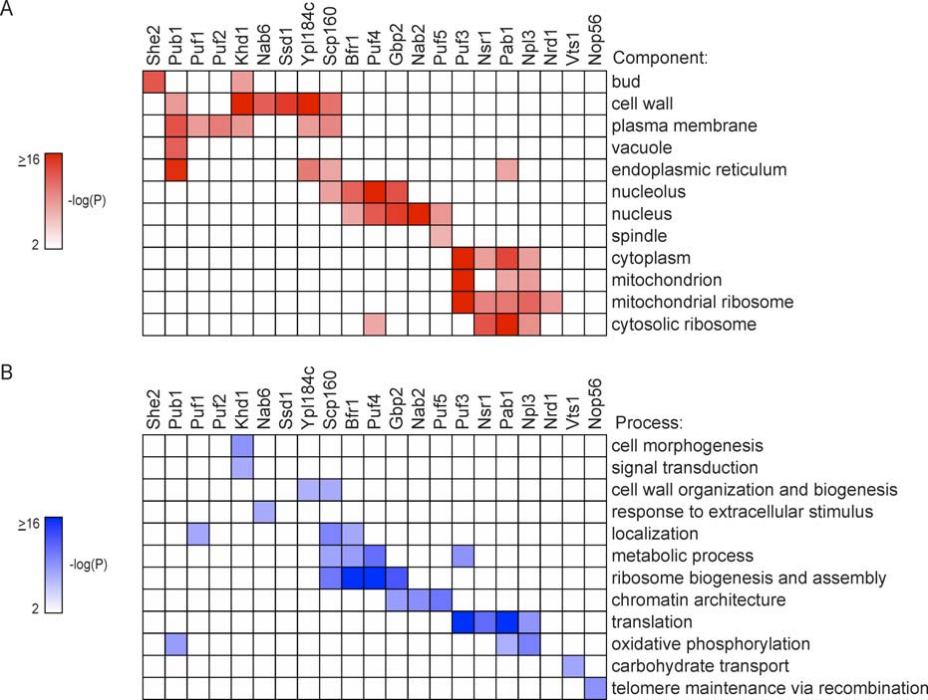
RNA-Binding Proteins Bind mRNAs Encoding Functionally and Cytotopically Related Proteins (A) Enrichment of “cellular component” GO terms (rows) in target sets (1% FDR) of RBPs (columns). The significance of enrichment of the GO term is represented as a heat map (scale is to the left of the figure) in which the color intensity corresponds to the negative log_10_ *p*-value, calculated using the hypergeometric density distribution function and corrected for multiple hypothesis testing using the Bonferroni method. Only a subset of significantly enriched GO terms are shown. RBPs whose targets are significantly enriched (*p* ≤ 0.01) for at least one “cellular component” or “biological process” GO term are shown. (B) Same as in (A), except for “biological process” GO terms.

Regulatory proteins, including both transcription factors and RBPs, typically regulate sets of targets that share identifiable functional relationships (e.g., [26–29,32,35,52–60]). As a first step toward identifying relationships among RNAs bound by specific RBPs, we searched for gene ontology (GO) terms [61] that were significantly enriched among the targets of each RBP. Twenty-five of the RBPs in this survey were consistently associated with at least ten mRNAs; 13 of these sets of RNA targets specific to an RBP were significantly enriched for at least one “cellular component” GO term (Figure 2A and Table S3), representing a shared subcellular localization or in some instances a protein complex, and 13 of these RBP-specific target sets were significantly enriched for at least one “biological process” GO term (Figure 2B and Table S3).

Diverse subcellular loci and biological processes were represented among the annotations enriched in the sets of RNA targets of these 15 RBPs (as well as the five Puf proteins and She2), including nearly all major subcellular compartments. Some subcellular sites and biological processes were found as shared attributes of the RNA targets associated with an unexpectedly large fraction of the RBPs in this study, perhaps highlighting processes or systems in which post-transcriptional regulation plays an especially important role. For instance, six RBPs (Pub1, Khd1, Nab6, Ssd1, Ypl184c, and Scp160) were specifically associated with mRNAs encoding cell wall proteins; six (Pub1, Puf1, Puf2, Khd1, Ypl184c, and Scp160) were specifically associated with mRNAs encoding plasma membrane proteins; five (Puf3, Nsr1, Pab1, Npl3, and Nrd1) were significantly associated with mRNAs encoding subunits of mitochondrial ribosome; and four (Scp160, Bfr1, Puf4, and Gbp2) were specifically associated with mRNAs encoding proteins localized to the nucleolus and involved in RNA processing and ribosome biogenesis.

For many RBPs, several distinct subcellular components or biological processes were overrepresented in the functional annotations of the associated transcripts; these subcellular loci or processes were often functionally linked. For example, RNAs associated with Ssd1 were enriched for transcripts encoding cell wall and bud proteins, whereas Gbp2-associated RNAs were enriched for transcripts encoding nuclear proteins with roles in ribosome biogenesis or chromatin remodeling. In many instances, the functional themes significantly overrepresented among the RNA targets of an RBP are congruent with previously published work on that RBP, such as phenotypes associated with mutation of altered expression (Table S2). A few examples are described in subsequent sections.

### Specific Features of Post-Transcriptional Regulation May Be Linked to Broad-Specificity RNA-Binding Proteins

Although some appear to bind to most or all mRNAs (Figure S2 and Text S3), the nine RBPs that bind large (>500) sets of mRNAs display several distinct enrichment profiles (Figure 1B), with correspondingly different GO annotations overrepresented among the most highly enriched mRNAs (Figure 2). In addition, for each of these nine RBPs, immunoaffinity enrichment of mRNAs with the RBP was significantly correlated with either ribosome occupancy [62], abundance [19], half-life [19], 3′-UTR length [63], 5′-UTR length [63], mRNA length [63], coding sequence length, or in some cases, with more than one of these features (Figure S3). Quantitative differences in the enrichment of mRNAs in association with a given RBP could result from the number or affinity of the RBP molecules bound or differences in the fraction of its lifespan that an individual mRNA spends at the specific stage during which a particular RBP plays a role (Text S5).

Pab1 provides a simple and useful example of the possible functional significance of the differential enrichment; immunoaffinity enrichment of mRNAs associated with Pab1 was correlated with ribosome occupancy (Pearson correlation = 0.35). Pab1 is the major poly(A) binding protein in both the nucleus and cytoplasm [64]. In the cytoplasm, Pab1 binds to the poly(A) tails of mRNAs and interacts with eIF4-G to promote translation initiation [65]. Because longer poly(A) tails have been reported to increase translation efficiency [66], a possible interpretation of these results is that the observed enrichment could reflect the number of Pab1 proteins bound per mRNA and thus the length of the poly(A) tail [39].

In contrast, immunoaffinity enrichment with Khd1 was negatively correlated with ribosome occupancy (*r* = −0.26). Khd1 is implicated in repressing translation of ASH1 mRNA during the transport of the mRNA to the bud tip [67]. The negative correlation with global ribosome occupancy and the large number of mRNAs associated with Khd1 suggest that Khd1 may similarly repress translation initiation of hundreds to thousands of mRNAs, perhaps during their transport to specific cellular loci.

### Many RNA-Binding Proteins Appear to Bind Their Targets during Specific Stages in Their Lives

Many RBPs associate with mRNAs at a particular stage in their lives [2]. For the approximately 270 intron-containing genes, the relative enrichment of introns (i.e., unspliced pre-mRNAs and possibly uncleaved excised introns) versus exons (i.e., mature mRNAs and pre-mRNAs) should reveal whether the RBP is bound specifically to intron-containing transcripts, mature mRNAs, or both, and thus indicate when and where the RBP associates with its target RNAs. Linking these data to functional information on the RBP could then provide insights into timing and duration of specific stages in the lives of mRNAs.

**Figure 3:**
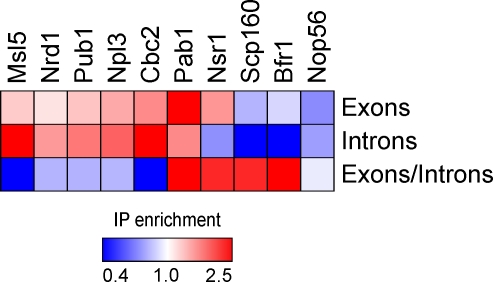
Differential Exon/Intron Association Suggests That Certain RNA-Binding Proteins Bind Their Targets during Specific Stages in Their Lives The relative enrichment of exons and introns in association with RBPs (columns) is represented using a color scale. Results are shown for RBPs that associated substantially more or less strongly with exons or introns than with RNAs overall (mean enrichment of exons from intron-containing genes or introns 25% above or below the median IP enrichment of all RNAs, respectively).

To test this idea, we compared the enrichment of intron and exon sequences in association with RBPs. For the approximately 120 intron/exon probe pairs for which our data were most consistently reliable, the relative enrichment profiles vary greatly among RBPs (Figure 3 and Text S6). For example, Cbc2 (a component of the heterodimeric nuclear cap-binding protein) and Pab1 were preferentially associated with both intron-containing transcripts and mature mRNAs derived from intron-containing transcripts (Figure 3). Cbc2 was strongly associated with intron-containing transcripts (mean enrichment of intronic sequences = 6.8), and also, but to a considerably lesser extent, with exon sequences from intron-derived transcripts (mean enrichment of exonic sequences = 1.5). These results are consistent with Cbc2 binding during transcription, prior to splicing, and being displaced shortly after the mature mRNA reaches the cytoplasm [68,69]. The enrichment of intron-related transcripts and the paucity of significantly enriched mature mRNAs suggest that most mRNAs spend only a very small fraction of their lives in the nucleus. That Pab1, the major poly(A) binding protein, associated with intron-containing transcripts (mean enrichment of intronic sequences = 1.5), as well as sequences from exons (mean enrichment of exonic sequences = 3.9), is consistent with most splicing occurring after poly(A) tail addition [70].

### Combinatorial Interactions among RNA-Binding Proteins and mRNAs

The RBPs we analyzed bound overlapping sets of mRNAs, and many individual mRNAs were bound by more than one RBP (Figure 1B and 1C). This network of interactions could support a robust and multidimensional regulatory program.

**Figure 4:**
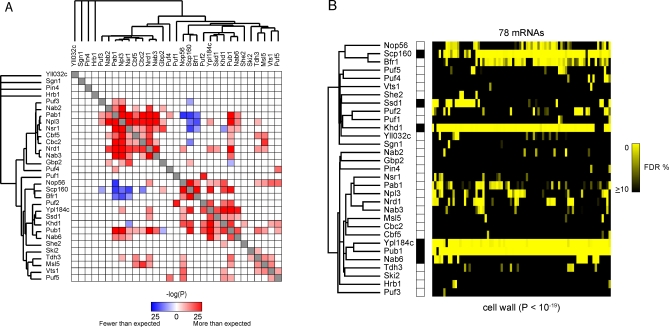
Combinatorial Interactions among RNA-Binding Proteins and mRNAs (A) The significance of the overlap between mRNA targets for each pair of RBPs (1% FDR threshold) is represented as a hierarchically clustered heat map in which the color intensity represents the negative log_10_ *p*-value, which was calculated using the hypergeometric density distribution and corrected for multiple hypothesis testing using the Bonferroni method. (B) An example of a cluster of functionally and cytotopically related mRNAs defined by their pattern of binding to multiple RBPs. The heat map represents RBPs (rows) and mRNAs (columns) color coded to reflect the certainty with which we infer that a specific mRNA is a target of a specific RBP (10% FDR [black] to 0% FDR [yellow]). These 78 mRNAs were associated (at a 1% FDR threshold) with at least four of a set of six RBPs (Ssd1, Khd1, Pub1, Ypl184c, Scp160, and Nab6) whose targets are enriched for mRNAs encoding proteins localized to the cell wall.

To explore the relationships among the groups of RNAs bound by different RBPs, we determined the extent to which the overlaps between targets for each RBP pair differed from what would be expected by chance. The significance values from this analysis were used as a metric of similarity for hierarchical clustering to identify pairs and sets of RBPs with similar patterns of shared targets. The results are presented in Figure 4A as a heat map, in which the similarity between the target sets of each pair of RBPs is shown on a blue (significantly fewer shared targets than expected, *p* = 10^−25^) to white (*p* > 0.001) to red (significantly more shared targets than expected, *p* = 10^−25^) scale. At a *p*-value threshold of 0.001, 69 of 465 RBP pairs shared significantly more mRNA targets than expected by chance, whereas 11 RBP pairs shared significantly fewer mRNA targets than expected by chance. Several of the most significantly overlapping target sets belong to sets of RBPs that are known to physically interact, such as Scp160 and Bfr1 [50], Nrd1 and Nab3 [51], Nrd1/Nab3 and Npl3 [71], and Nrd1/Nab3 and Pab1 [72].

To further explore the interrelationships among RBPs and their mRNA targets, we used a supervised method to identify smaller subsets of mRNAs that shared interactions with several RBPs. We did this by selecting mRNAs bound by a common set of RBPs whose targets, in turn, were enriched for common GO terms (Figure 2).

The group of mRNAs, defined by interactions with at least four of a set of six RBPs (Pub1, Khd1, Nab6, Ssd1, Ypl184c, and Scp160), includes a significant excess of mRNAs encoding proteins localized to the cell wall (Figure 4B); indeed, 23 of the 78 mRNAs in this cluster encode cell-wall proteins (*p* < 10^−19^). This group also contains mRNAs that encode proteins that are secreted (5), localized to sites of polarized growth (4), or localized to the ER (14). It is important to recognize that the unifying theme in this group is not narrowly restricted to simple functions in cell-wall metabolism—many mRNAs in this group encode proteins with diverse roles in regulation of cell-wall metabolism. Fifteen mRNAs encode proteins involved in post-transcriptional regulation, including SSD1, DHH1, and PUF5, which are genetically implicated in cell-wall biogenesis and maintenance [73,74], and NGR1 and WHI3, which are involved in control of cell growth [75–77]. Fourteen of these mRNAs encode proteins involved in transcriptional control, including SFL1, which is implicated in cell-wall assembly [78], and NDD1, YOX1, and NRM1, which are involved in cell-cycle control [79–81]. Seven mRNAs encode signal transduction proteins, including MFA2, CLN2, GIC2, WSC2, and MSB2, which are implicated in cell-wall growth or cell-cycle regulation [82–88].

### How Do the RNA-Binding Proteins Identify Their Targets?

We identified candidates for the sequence elements that mediate regulatory interactions with specific RBPs using two related computational methods: “finding informative regulatory elements” (FIRE), which searches for motifs with informative patterns of enrichment [89], and a newly developed method, “relative filtering by nucleotide enrichment” (REFINE). In brief, REFINE identifies all hexamers that are significantly enriched in putative 5′- and 3′-UTR regions of targets over nontargets, filters out regions of target sequences that are relatively devoid of such hexamers, and then applies the “multiple expectation maximization for motif elicitation” (MEME) motif-finding algorithm [90]. A full description of the REFINE methodology and more detailed analyses of predicted motif sequences will be published separately (D. P. Riordan, D. Herschlag, and P. O. Brown, unpublished data). Herein, we combined the results from these two approaches.

**Figure 5:**
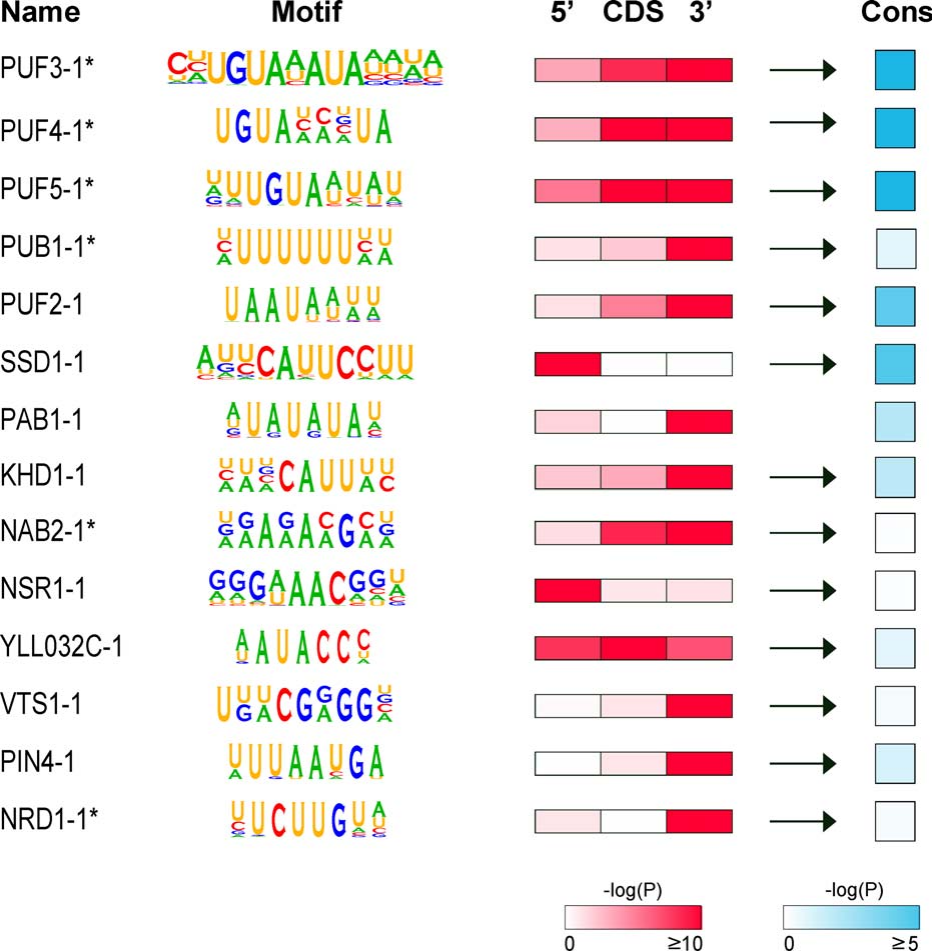
Diverse Sequence Motifs Enriched in mRNAs Bound by Specific RNA-Binding Proteins A pictogram (http://genes.mit.edu/pictogram.html) represents the regular expression patterns defined for FIRE motifs or the preferred base composition of the position-specific scoring matrices for REFINE motifs. For each motif, the negative log_10_ *p*-value of the significance of genome-wide enrichment for motif sites in targets is represented (using a color scale) for segments of its mRNA targets (5′ = 200 bases upstream of start codon, CDS = protein coding sequence, 3′ = 200 bases downstream of stop codon). Arrows indicate motifs with a forward strand bias, i.e., the reverse complements of the motifs are not significantly enriched (*p* > 10^−4^ based on the hypergeometric distribution) in targets. “Cons” indicates the negative log_10_ *p*-value measuring whether motif sites in targets are more likely to be conserved in orthologous sequence alignments in S. bayanus than are motif sites occurring in nontargets, based on the hypergeometric distribution. Asterisks (*) denote motifs matching previously described RNA-binding elements (details in text). Exact data values and full descriptions of all motifs are presented in Table S4.

Using stringent statistical criteria based on randomized simulations (details in Materials and Methods), we identified a total of 60 candidate RNA regulatory motifs significantly associated with 21 different RBPs; 35 motifs (for 21 RBPs) were predicted by REFINE, and 25 motifs (for 13 RBPs) were predicted by FIRE (Table S4). Since the same motifs were often predicted by both programs for the same RBP or for different RBPs with significantly overlapping target sets, we manually grouped motifs with similar consensus sequences and origins into classes (Table S4). We then included only the most significant motif from each class and for each RBP, resulting in a set of 14 nonredundant RNA motifs predicted with high confidence (Figure 5). We also evaluated the predicted RNA motifs by testing whether motif sites occurring in targets were more likely to be conserved than sites in nontargets, and whether they exhibited a forward strand bias by testing for significant enrichment of the reverse complementary motif in RBP targets (Table S4).

The motifs we identified for Puf3, Puf4, Puf5, Pub1, Nab2, Nrd1, and Nab3 match previously described binding sites for the corresponding RBPs, validating our approach and suggesting that many of the RBP–RNA interactions we measured are likely to be directly mediated by these elements (Text S7). Interestingly, the inferred recognition element for Nrd1, Nrd1–1 (UUCUUGUW), contains both an exact match to the reported Nrd1 binding site consensus “UCUU” and a partial match to the reported Nab3 recognition site consensus “GUAR” [91,92]. As Nrd1 and Nab3 are known to act as a complex to control transcriptional termination of nonpolyadenylated RNAs [93], and a nearly identical motif was identified in Nab3 targets (Table S4), it is possible that these motifs represent a favored orientation of adjacent Nrd1 and Nab3 RNA elements that facilitates specific binding of the Nrd1–Nab3 complex.

The most significant novel motif we identified, Puf2–1 (UAAUAAUUW), is enriched in the 3′-UTRs and coding sequences of Puf2 targets and demonstrates significant conservation and a forward strand bias (Figure 5). This motif is similar to a motif identified for the paralogous RBP Puf1, which associates with a subset of the Puf2 target mRNAs (Table S4). The next most significant novel motif, Ssd1–1 (AKUCAUUCCUU), is highly enriched in the 5′-UTRs of Ssd1 targets (Figure 5). Although its presence upstream of the coding sequences of Ssd1 target genes would also be consistent with a role as a transcription factor binding site, its tendency to occur within the annotated 5′-UTRs of targets (63% targets versus 19% nontargets, *p* < 10^−6^) [94], its dramatic enrichment in targets, and its forward strand bias suggest that this RNA motif is recognized by Ssd1.

**Figure 6:**
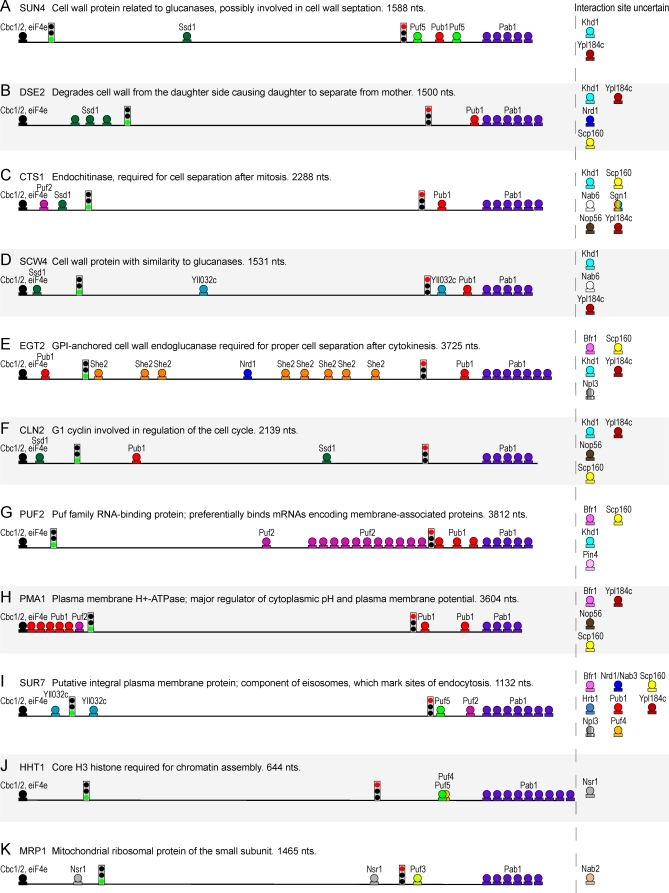
Diverse Combinatorial Patterns of RNA-Binding Protein Interactions with a Choice Sample of mRNAs (A–K) Putative binding sites of RBPs in target mRNAs. The relative lengths of the 5′-UTR, coding sequence, and 3′-UTR are drawn to scale. For mRNAs for which there are reliable measurements for untranslated sequence lengths (SUN4, DSE2, SCW4, CLN2, PUF2, PMA1, SUR7, and HHT1) [63], we added 50 bases onto the estimated 5′-UTR and 3′-UTR lengths, because the estimated UTR lengths are likely conservative. For mRNAs that do not have reliable untranslated region measurements (CTS1, EGT2, and MRP1), we used 250 bases upstream and downstream of the coding sequence as the 5′-UTR and 3′-UTR, respectively. The positions of the start and stop codons are indicated by stop signals. Putative binding sites for RBPs with strong evidence for association (1% FDR) are marked (Puf3-REFINE, Puf4-FIRE, Puf5-REFINE, Pub1-FIRE, Puf1/2-REFINE, Ssd1-REFINE, Nsr1-REFINE, Yll032c-REFINE, Pin4-REFINE, and Nrd1/Nab3-REFINE) (Figure 5 and Table S4). RBPs that we found to be associated with the mRNA, but for which the recognition elements are not yet known, are listed to the right of the mRNA. The number of Pab1 molecules shown bound to the poly(A) tail represents the degree of enrichment of the corresponding mRNA in the Pab1 IPs (log_2_ immunopurification enrichment −6 = 0, −5 = 1, etc.) and not the number of Pab1 molecules bound per mRNA. The cap-binding proteins, Cbc1/2 and eiF4e, are shown by default at the cap site.

A selective sample of 11 mRNAs provides an unfinished, but revealing, picture of the organization of the information that specifies interactions with, and perhaps regulation by, specific RBPs examined in this study (Figure 6). For each mRNA, the location of high-confidence RNA recognition elements for RBPs that interact with the mRNA are indicated, while RBPs that interact with the mRNA, but whose binding site is uncertain, are shown to the right of the mRNA. The relative lengths of the 5′-UTR, coding sequence, and 3′-UTR are drawn to scale, and the translation start and stop codons are depicted with the corresponding “traffic signal.” Each of these mRNAs has specific interactions with overlapping, but distinct, subsets of RBPs in the study. The putative binding patterns of specific RBPs, with respect to the number and locations of sites, vary considerably among the mRNAs, which may have important functional consequences. The first five mRNAs (SUN4, DSE2, CTS1, SCW4, and EGT2) encode cell-wall enzymes (Figure 6A–6E). Each of these mRNAs associated with five to nine RBPs in this study, including all five with Pub1, Khd1, and Ypl184c, four with Ssd1 (SUN4, DSE2, CTS1, and SCW4), three with Scp160 (CTS1, SCW4, and EGT2), and two with Nab6 (CTS1 and SCW4) and Nrd1 (DSE2 and EGT2). In addition to these overlapping interactions, most of these mRNAs associated with a unique set of additional RBPs; for instance, SUN4 contains two Puf5-binding sites in its 3′-UTR and EGT2 contains eight She2-binding sites in its coding sequence. CLN2 encodes a G1 cyclin and associated with many of the same RBPs as SUN4, DSE2, CTS1, SCW4, and EGT2 (Figure 6F). PUF2 associated with several RBPs, including its cognate protein, which is common among RBPs in this study (Text S8); there are 12 Puf2-binding sites in its coding sequence (Figure 6G). PMA1 associated with a similar set of RBPs as PUF2, including Pub1 and Puf2, but the locations and numbers of binding sites for these RBPs are very different in the two mRNAs (Figure 6H). The putative binding sites for Puf4 and Puf5 in the 3′-UTR of HHT1 partially overlap, suggesting these RBPs may compete for binding to this mRNA (Figure 6J). These diagrams represent only a partial picture of the RBP interactions with these mRNAs; the mRNA targets have only been defined for a small fraction of all yeast RBPs, and the sequence elements that specify many of the interactions we have identified are not yet known.

For many RBPs, our computational method did not identify any sequence motifs with statistically significant enrichment, the motifs identified significantly overlapped those associated with other RBP target sets, or the motif did not match previously reported binding preferences (Table S4 and Text S7). The large degree of motif coenrichment observed in our analysis is consistent with combinatorial regulation by a highly interconnected regulatory network and represents an important limitation of computational regulatory element identification. It is likely that some of the RBPs for which we failed to predict sequence motifs recognize RNA structural elements or features primarily present in coding sequences, which are difficult to detect with current methods for RNA motif prediction, because they are not suited to modeling structural features or handling the significant confounding sequence biases in coding sequences.

Vts1 illustrates some of the limitations of current RNA motif prediction methods. Vts1 is known to bind to a structural RNA motif called the Smaug recognition element (SRE), which consists of a short hairpin with the loop consensus sequence CNGGN(0–1) [95]. SRE sites are indeed significantly enriched in the coding sequences of Vts1 targets (65% targets versus 36% nontargets, *p* < 10^−7^) in agreement with previous results [96], suggesting that SRE elements are directly responsible for these interactions in vivo. However, neither REFINE nor FIRE succeeded in identifying the SRE. Instead, both programs identified a motif, Vts1–1 (UKWCGRGGN), which is indeed enriched in the 3′-UTRs of Vts1 targets but is unrelated to the SRE (Table S4). We suspect that the Vts1–1 motif may represent a binding site for an unknown factor that regulates a set of mRNAs that overlaps extensively with the targets of Vts1.

It is likely that direct high-resolution mapping of in vivo RBP binding sites and systematic in vitro characterization of binding preferences of RBPs will overcome some of the limitations in current methods for RNA motif identification [97,98].

### Insights into the Functions of Specific RNA-Binding Proteins

The functional and cytotopic themes represented among the specific targets of each RBP have obvious implications for their possible regulatory roles, which can be integrated with previously reported information to derive further insights, and generate new hypotheses, as illustrated here for Ssd1 and Ypl184c (see Text S9 for descriptions of Khd1 and Gbp2).

Ssd1 is a large (140 kDa), ribonuclease-II domain–containing, predominantly cytoplasmic protein [99], genetically implicated in cell-wall biogenesis and function: mutant phenotypes include increased sensitivity to osmotic stress and caffeine, altered composition and structure of the cell wall, defects in germination and sporulation, premature aging, and pathogenicity [73,74,100–103]. Ssd1 physically and genetically interacts with numerous signaling proteins, many of which are genetically implicated in cell-wall function [71,102,104,105]. Ssd1 binds to the C-terminal domain of RNA polymerase II in vitro [106].

Of the 52 annotated mRNAs associated with Ssd1, 16 encode proteins localized to the cell wall (*p* < 10^−15^), and 11 encode proteins localized to the bud (*p* < 10^−5^). The proteins encoded by the Ssd1-associated transcripts have diverse functional and structural roles related to cell-wall biosynthesis, or remodeling and its regulation, cell-cycle progression, and protein trafficking. Ssd1 also appears to bind its own transcript (Text S8).

For both of the Ssd1 mRNA targets encoded by intron-containing genes (PUF5 and ECM33), the intron-containing primary transcripts are also enriched by Ssd1 IP, suggesting that Ssd1 binds its RNA targets in the nucleus, perhaps while they are being transcribed. A putative RNA-recognition motif is significantly enriched in the 5′-UTRs of Ssd1 targets (Figure 5). The numbers and positions of this motif in Ssd1-bound RNAs vary widely among its targets (Figure 6A–6D and 6F). These data lead us to speculate that Ssd1 binds its targets cotranscriptionally by recognizing a specific RNA motif and prevents their translation initiation until these mRNAs reach specific locations in the cell, such as the ER membrane, bud, or sites of cell-wall biosynthesis. The multiple phosphorylation sites on Ssd1 could regulate the localization, binding, and release of its RNA targets. Although Ssd1 is a ribonuclease-II domain–containing protein, it has no discernable nuclease activity [99]. Given that Ssd1 does not contain any other known RNA-binding domains, we suggest that the ribonuclease-II domain may have evolved into a sequence-specific RNA-binding domain in this protein family.

Ypl184c is a largely uncharacterized, predominantly cytoplasmic protein that contains three RNA recognition motifs (RRMs). Of the three proteins that have been found to physically interact with Ypl184c, two are among the other RBPs included in this survey: Pab1 and Nab6 [71].

A disproportionate fraction of the 321 annotated mRNAs we found to associate with Ypl184c encode proteins localized to the cell wall (38, *p* < 10^−23^), ER (50, *p* < 10^−5^), plasma membrane (32, *p* < 10^−3^), or extracellular milieu (8, *p* < 10^−3^). Transcripts encoding components of several protein complexes were associated with Ypl184c, including three of five components of the Cdc28 complex (CLB2, CLN3, and CLN2) for which we obtained high-quality measurements, three of three components of the plasma membrane H^+^ ATPase (PMP1, PMP2, and PMA1) for which we obtained high-quality measurements, and four of nine components of the oligosaccharyltransferase complex (OST4, SWP1, OST3, and OST5) [107]. Components of these complexes that were not defined as targets of Ypl184c (at a stringent 1% FDR) were nevertheless more likely to be overrepresented in Ypl184c IPs than expected by chance, suggesting that Ypl184c may actually associate with the mRNAs encoding most or all members of these complexes.

Ypl184c associated with many mRNAs that exhibit unusual modes of translation regulation. Ypl184c bound all five of the mRNAs that have experimentally confirmed short upstream open reading frames (uORFs) (GCN4, CPA1, LEU4, SCH9, and SCO1) [108–115] in their 5′-UTRs and for which we obtained high-quality measurements; uORFs have been shown to regulate the translation of the downstream coding sequence and the stability of the mRNA [116]. Ypl184c associated with all five of the S. cerevisiae mRNAs that have been shown to have internal ribosome entry sites (IRES) (HAP4, YMR181C, GPR1, NCE102, and GIC1) in their 5′-UTRs [117,118] for which we obtained high-quality measurements; these IRESs enable cap-independent translation, often in response to environmental stresses [119]. Ypl184c also bound the unspliced HAC1 transcript, which associates with the cytosolic side of the ER membrane and is not efficiently translated until it is spliced by IRE1 as part of the unfolded protein response pathway [120,121].

Given Ypl184c's association with Pab1 and its striking association with sets of mRNAs that are known to be subject to extensive translational regulation, we speculate that Ypl184c regulates translation. The sequence motifs that we found to be significantly enriched in the mRNA targets of Ypl184c closely match the ones we found for Pub1 (Table S4). Indeed, the RNA target sets of these two proteins overlap significantly (Figures 1B and 4A). Given the absence of evidence for direct interactions between Ypl184c and Pub1, perhaps they compete for binding to overlapping groups of mRNAs. We have named YPL184C, post-transcriptional regulator of 69 kDa (PTR69).

## Discussion

A large body of work has given us a general picture of the relationship between the several hundred transcription factors and thousands of genes in yeast (e.g., [26–29,32,35,52–60]). Among the key features of transcriptional regulation are that: (1) individual transcription factors characteristically regulate sets of genes with related biological roles, (2) transcription factors are recruited to the specific genes they regulate by binding to specific sequences in the vicinity of those genes, and (3) combinatorial regulation of individual genes by two or more distinct transcription factors provides multidimensional control and precision to their regulation. Our systematic identification of RNAs associated with each of 46 proteins in yeast suggests that a system that shares these three key features, likely involving dozens to hundreds of RBPs, may regulate the post-transcriptional fate of most or all RNAs in the yeast cell.

This glimpse into the landscape of RNA–protein interactions has provided tantalizing clues to its organization and role. The mRNA targets of most of the RBPs in the survey encoded sets of proteins that were significantly associated with one or several related subcellular sites or biological processes (Figure 2 and Table S3). Although the regulatory roles and molecular mechanisms of most of these interactions remain to be elucidated, it seems unlikely that they have a purely decorative function. The selective binding of RBPs to sets of mRNAs that encode functionally and cytotopically related proteins provides strong evidence for widespread regulation at the post-transcriptional level. The functional relevance of these interactions is further supported by their relationships to phenotypes associated with mutation or altered expression of the RBP (Table S2). Many RBPs, including those examined in our survey, have mutant phenotypes only in specific physiological and developmental programs, and they have diverse gene expression patterns (http://www.yeastgenome.org). Thus, the regulatory program mediated by RBPs may be reorganized in response to specific physiological and developmental cues.

The striking tendency of individual RBPs to bind to sets of mRNAs whose protein products are similarly localized in the cell hints at an important role for RBPs in establishing and maintaining spatial organization in the cell, perhaps through facilitating localized protein production and mRNA decay [13,32,122–131]. The cellular structures that were most often overrepresented among the mRNA targets of many RBPs were the cell wall, plasma membrane, and ER. Thus, in addition to the familiar role of the peptide signal sequence in mediating ER-localized translation [12], RBPs may have important roles in RNA partitioning between the cytoplasm and ER, and perhaps in localization to specific sites in the periphery of the cell, such as sites of cell-wall biogenesis, bud development, and endocytosis [32,132–135]. Two of the RBPs whose targets disproportionably encode proteins localized to the cell periphery, She2 and Khd1, have been shown to be involved in trafficking some of their mRNA targets to the bud tip during the G2/M phase of the cell cycle [32,67,136]. The particularly strong overrepresentation of RBPs that associate with mRNAs encoding cell-wall components may reflect the need for extensive multilayered regulation of the location and timing of assembly and remodeling of this dynamic subcellular structure.

Identification of the information that specifies mRNA–RBP interactions is still in its earliest stages. The sequence motifs overrepresented in RBP targets, identified with the recently developed FIRE and novel REFINE methodologies, are diverse in design and location (Figures 5 and 6). Many of these RBPs recognized short linear sequences in the 3′-UTRs, 5′-UTRs, or coding sequences, or two or more of these regions. For about half of the RBPs, however, we were unable to find a sequence motif enriched among its RNA targets. Some of these RBPs may recognize structural elements. In support of this idea, we found the SRE hairpin loop, previously recognized as important for specific recognition of RNA by Vts1 [95], significantly enriched in coding sequences of Vts1 targets. Another protein in this survey, She2, is believed to recognize a three-dimensional structure in its targets [137,138]. We found promoter elements that likely specify transcription factor interactions enriched in the upstream regions of several RBP target sets, e.g., Gbp2 (Table S4). It is possible these promoter elements play an indirect role in specifying RBP interactions, perhaps by cotranscriptional recruitment of an RBP to mRNA targets via interactions with specific transcription-associated factors [22,23,139]. Identification of the large amount of still-undiscovered RNA regulatory information is an essential step in uncovering the specific regulatory program of each gene.

We identified over 12,000 mRNA–RBP interactions with high confidence. Most mRNAs in the yeast transcriptome associated with at least one of the RBPs in our survey and many associated with multiple RBPs. Some of the RBPs in the survey appear to interact with most or all mRNAs at some point in their lifecycle (Figure S1 and Text S3). Naively extrapolating from our results to the estimated 600 RBPs in *Saccharomyces* suggests that each mRNA might interact with a dozen or more different RBPs, on average, during its lifetime. This extrapolation is highly speculative; the sample of RBPs that we investigated is biased towards RBPs that we suspected might have a regulatory function; we do not have a good estimate of the number of regulatory RBPs that bind discrete sets of mRNAs in the manner analogous to specific transcription factors; given that three of the four proteins in this survey that were not annotated as RBPs nevertheless gave reproducible interactions with specific sets of mRNAs (Bud27, Aco1, and Tdh3), the number of potential noncanonical, unannotated RBPs with regulatory roles may be large, perhaps even in the hundreds [140–144].

There is no reason to believe the system we have described is peculiar to yeast. Extensive post-transcriptional regulation by combinatorial binding of a large and diverse set of specific RBPs is likely to be a general feature of regulation in eukaryotes. Indeed, several lines of evidence suggest an even greater genomic investment in post-transcriptional regulation in humans (and other metazoans); the number and diversity of RBPs encoded by the human genome seems to far exceed that of yeast [145], untranslated regions of mRNAs are much longer in humans (∼1,300 bases on average) than in yeast (∼300 bases on average) and appear to contain much more regulatory information [6,146,147], and the architecture of animal cells is far more diverse and complex than that of the yeast cell, with a correspondingly greater potential role for specific RNA localization [13,130,148–151].

This work has provided a glimpse of a network of RBP–mRNA interactions that is likely to play an important, but still largely undiscovered, role in biological regulation. The genes and *cis*-regulatory elements implicated in this process represent a substantial fraction of the genome's investment in regulation, yet the specific details and molecular mechanisms of this network of RBP–mRNA interactions are still largely terra incognita—and fertile ground for further exploration and discovery.

## Materials and Methods

### RNA imunoaffinity purifications.

We carried out immunopurifications of specific proteins, together with the associated RNAs, using specific strains expressing a TAP-tagged derivative of each selected protein (Open Biosystems Cat# YSC1177-OB), essentially as described in Gerber et al. [26]. After growing 1L cultures to an optical density at 600 nm (OD_600_) of 0.6–0.9 in YPAD, we harvested cells by centrifugation, chilled the cell pellets on ice, washed them twice with 25 ml of ice cold buffer A (20 mM Tris–HCl [pH 8.0], 140 mM KCl, 1.8 mM MgCl_2_, 0.1% Nonidet P-40, 0.02 mg/ml heparin), then froze them in LN_2_ and stored them at −80 °C. In a few instances, we proceeded to lyse the pelleted cells immediately without freezing. To lyse the cells, we first thawed the cell suspension at 4 °C, added 5 ml of buffer B (buffer A plus 0.5 mM DTT, 1 mM PMSF, 1 μg/ml leupeptin, 1 μg/ml pepstatin, 20 U/ml DNase I [Stratagene Cat# 600032], 50 U/ml Superasin [Ambion Cat# AM2696], and 0.2 mg/ml heparin), and then mechanically lysed the cells by vortexing in the presence of glass beads. We removed the beads by centrifugation at 1,000*g* for 5 min, then clarified the extracts by centrifuging them twice at 7,000*g* for 5 min each. We adjusted the volume of the extract to 5 ml with buffer B, removed a 100-μl aliquot for reference RNA isolation, and then incubated the remaining 4.9 ml with 400 μl of 50% (v/v) suspension of IgG-agarose beads (Sigma Cat# A2909) in Buffer A with gentle rotation for 2 h. We washed the beads once with 5 ml of buffer B for 15 min, and three times with 12 ml of buffer C (20 mM Tris-HCl [pH 8.0], 140 mM KCl, 1.8 mM MgCl_2_, 0.5 mM DTT, 0.01% NP-40, 15 U/ml Superasin, 1 μg/ml pepstatin, 1 μg/ml leupeptin, 1 mM PMSF) for 15 min with gentle rotation. We pelleted the beads by centrifugation for 5 min at 60*g* in a table-top centrifuge. We then transferred the beads to 1.2-ml micro-spin columns (BioRad Cat# 732-6204), centrifuged them briefly to pellet the beads, removed buffer C, and then added 1 volume of buffer C. We cleaved TAP-tagged proteins by incubation with 80 U acTEV protease (Invitrogen Cat# 12575023) or an equivalent amount of purified TEV [152] for 2 h at 15 °C. We collected the eluent by centrifugation into 2-ml tubes. We isolated reference RNA using RNeasy Mini Kit (Qiagen Cat# 74106), while we isolated RNA from the eluate by extraction with Phenol/Chloroform/Isoamyl Alcohol, 25:24:1 (Invitrogen Cat# 15593031) twice, and chloroform once, followed by ethanol precipitation with 15 μg of Glycoblue (Ambion Cat# AM9515) as carrier.

### Oligonucleotide microarray design.

Starting with the Operon AROS 1.1 oligo set, which contains long oligonucleotides for almost all annotated S. cerevisiae nuclear and mitochondrial coding sequences, we added 3,072 additional probes designed to detect annotated noncoding RNAs, ribosomal RNA precursors, introns, exon-intron and exon-exon junctions, other sequences predicted to be expressed, additional probes for genes with high cross-hybridization potential, and hundreds of controls for array quality measurements and normalization. Details of oligonucleotide selection and probe sequences are available from the Operon Web site (https://www.operon.com/; S. cerevisiae YBOX V1.0).

### Microarray production and prehybridization processing.

Detailed methods for microarray experiments are available at the Brown lab Web site (http://rd.plos.org/pbio.0060255).

For oligonucleotide microarrays, we resuspended oligonucleotides in 3× SSC (1× SSC = 150 mM NaCl, 15 mM sodium citrate [pH 7.0]) at a final concentration of 25 μM and printed oligonucleotides on poly-lysine glass (Erie Scientific Cat# C41–5870-M20) (http://rd.plos.org/pbio.0060255a). We printed each oligonucleotide twice per array. For most arrays, the second print was in reverse orientation to the first print, such that oligonucleotide pairs were printed with different pins and thus located in different sectors of the array.

Prior to hybridization, the oligonucleotides were crosslinked to the poly-lysine–coated surface with 65 mJ of UV irradiation. Slides were then incubated in a 500-ml solution containing 3× SSX and 0.2% SDS for 5 min at 50 °C. Slides were washed for 2 min in a glass chamber containing 400 ml of water, dunked in a glass chamber containing 400 ml of 95% ethanol for 15 s, and then dried by centrifugation. Free poly-lysine groups were then succinylated by incubation with 5.5 g of succinic anhydride that was dissolved in 350 ml of anhydrous 1-methyl,2-pyrolidoinone (Sigma Cat# 328634) and 15 ml of 1 M sodium borate (pH 8.0) for 20 min [53]. Slides were washed for 2 min in a glass chamber containing 400 ml of room temperature water, dunked in a glass chamber containing 400 ml of 95% ethanol for 15 s, and then dried by centrifugation.

cDNA microarrays containing long double-stranded DNA (dsDNA) from PCR reactions were prepared as previously described [53].

### Microarray sample preparation, hybridization, and washing.

A total of 3 μg of reference RNA from extract and up to 3 μg (or 50%) of affinity-purified RNA were reverse transcribed with Superscript II (Invitrogen Cat# 18064–014) in the presence of 5-(3-aminoallyl)-dUTP (Ambion Cat# AM8439) and natural dNTPs (GE Healthcare Life Sciences Cat# US77212) with a 1:1 mixture of N9 and dT20V primers (Invitrogen). Subsequently, amino-allyl–containing cDNAs were covalently linked to Cy3 and Cy5 NHS-monoesters (GE Healthcare Life Sciences Cat# RPN5661). Dye-labeled DNA was diluted in a 20–40-μl solution containing 3× SSC, 25 mM Hepes-NaOH (pH 7.0), 20 μg of poly(A) RNA (Sigma cat # P4303), and 0.3% SDS. The sample was incubated at 95 °C for 2 min, spun at 14,000 rpm for 10 min in a microcentrifuge, and then hybridized at 65 °C for 12–16 h. For most oligonucleotide microarray experiments, we hybridized microarrays inside sealed chambers in a water bath using the M-series lifterslip to contain the probe on the microarray (Erie Scientific Cat # 22x60I-M-5522). For some oligonucleotide microarray experiments, we hybridized microarrays using the MAUI hybridization system (BioMicro), which promotes active mixing during hybridization. We hybridized cDNA microarrays inside sealed chambers in a water bath using a coverslip to contain the probe on the microarray.

Following hybridization, microarrays were washed in a series of four solutions containing 400 ml of 2× SSC with 0.05% SDS, 2× SSC, 1× SSC, and 0.2× SSC, respectively. The first wash was performed for 5 min at 65 °C. The subsequent washes were performed at room temperature for 2 min each. Following the last wash, the microarrays were dried by centrifugation in a low-ozone environment (<5 ppb) to prevent destruction of Cy dyes [153,154]. Once dry, the microarrays were kept in a low-ozone environment during storage and scanning (see http://rd.plos.org/pbio.0060255).

### Microarray scanning and data processing.

Microarrays were scanned using either AxonScanner 4200, 4000B, or 4000A (Molecular Devices). PMT levels were adjusted to achieve 0.1%–0.5% pixel saturation. Each element was located and analyzed using GenePix Pro 5.0 (Molecular Devices). These data were submitted to the Stanford Microarray Database [155] for further analysis. Data were filtered, as described in Text S10, to remove low-confidence measurements. Oligonucleotide pairs that both passed filtering criteria were averaged, and the data were globally normalized per array such that the mean log_2_ (Cy5/Cy3 fluorescence) ratio was zero after normalization. We analyzed a total of 123 IPs by microarray hybridization (Dataset S1). During the course of this work, we continued to improve and optimize our protocols. These changes and the manufacturing differences in reagents (especially in the beads used in the IPs) led to systematic differences in the background distribution of RNAs between corresponding experiments. We minimized systematic differences among sets of experiments by deriving estimates of the background separately for each set of experiments. Each group was normalized by subtracting the median log_2_ ratio for each molecular features across the experiments in a group from the log_2_ ratio of the molecular feature in each experiment. The details of the group normalization are described in Text S10, and the groups are labeled in Table S5.

### Microarray analyses.

Hierarchical clustering was performed with Cluster 3.0 [156], and the results were visualized as heat maps with Java TreeView 1.0.12 [157]. Clustering of FDR values (Figures 1B and 4B) was performed using the centered Pearson correlation as a similarity metric. FDR values that were greater than or equal to 10 and missing values were set to 10 prior to clustering. Clustering of the significance values measuring the degree of overlap between RBP target sets (Figure 4A) was performed using the uncentered Pearson correlation as a similarity metric.

For SAM, unpaired two-class *t*-tests were performed with default settings. FDRs were generated from up to 1,000 permutations of group normalized data. Details of SAM analysis are described in Text S11.

### Enrichment of specific gene lists in RBP target sets.

The *p*-values of enrichment of specific classes of RNAs and GO terms in target sets were determined using the hypergeometric density distribution function and corrected for multiple hypothesis testing using the Bonferroni method. Enrichment of GO terms was performed with GO::TermFinder [158]. For noncoding RNAs, all RNAs for which we obtained reliable measurements on the microarray were used as background. For GO analysis, only probes that are meant to capture mature mRNAs were included in analyses. For oligonucleotide microarray experiments, this corresponds to probes that match the following regular expression: Y[A-P][RL][0–9]{3}[WC][-ABC]*_ORF (Datasets S1–S3). For cDNA microarray experiments, this corresponds to probes that match the following regular expression: Y[A-P][RL][0–9]{3}[WC][-ABC]* (Datasets S1–S3). mRNAs for which we obtained high-quality measurements were used as background.

### Sequences used for motif analysis.

Yeast sequence files orf_genomic_1000.fasta and orf_coding.fasta were downloaded from SGD (ftp://ftp.yeastgenome.org). The 200 nucleotides upstream and downstream of coding sequences containing proper start and stop codons were extracted to create 5′-UTR and 3′-UTR databases, and the coding sequences were used for the coding sequence database. All-by-all WU-BLAST [159] (http://blast.wustl.edu/) comparisons were performed for each database against itself to identify highly similar sequences (using options -e 1e-10 -b 5000 -S 1 -F F). WU-BLAST output files were parsed to identify alignments of greater than or equal to 80% identity extending over half the length of the query sequence, and all such sequence pairs were grouped into redundant classes. One sequence from each redundant class was retained to create nonredundant databases for each region.

### Motif prediction.

The REFINE procedure was run using hexamers with significant (*p* < 10^−3^) enrichment in RBP targets, as measured by the hypergeometric distribution (using options –ss –f 3 –g 6 –ct 3 –max 15 –dust). MEME analysis (version 3.5.1) was performed on the REFINE output sequences with options –dna –minw 6 –maxw 15 –text –maxsize 200000 –evt 10 –nmotifs 3. Motif site sequences were extracted from MEME output and used to generate position-specific log-odds scoring matrices based on the observed frequencies and 0.25 pseudocounts per base, and null frequencies based on mononucleotide composition of all sequences in the corresponding (5′-UTR or 3′- UTR) nonredundant database. Cutoff scores for motif classification were chosen to maximize the significance of association of motif sites with RBP target membership as measured by hypergeometric *p*-values for enrichment. All subsequences with scores above the cutoff threshold were classified as motif sites, and the final significance was measured as the negative log of the *p*-value of motif enrichment in RBP targets. FIRE analysis was run on the nonredundant 5′- and 3′-UTR databases using binary data indicating RBP target membership with options –exptype=discrete –seqlen_rna=200 –nodups=1 –dodna=0.

### Simulations to evaluate significance of predicted motifs.

For both REFINE and FIRE, statistical significance of the predicted motifs was assessed by randomly generating target sets of similar size and repeating each procedure 100 times on the simulated target data. We defined a test statistic as the negative log of the *p*-value for motif enrichment for REFINE; the reported motif *z*-score was used for FIRE motifs, and we compared the observed values of these test statistics to the distributions generated by the random simulations (Table S4). Motifs were declared as significant if the observed test statistic was greater than three standard deviations above the mean, or if there was significant enrichment (*p* < 10^−4^) of the motif in targets occurring in regions from which that motif was not derived.

## Supporting Information

Dataset S1 Normalized Data from DNA Microarray Experiments; Values from Both Pregroup Normalization and after Group Normalization Are Included (6.76 MB ZIP)

Dataset S2 Data Matrix Containing False-Discovery Rate Values for Each RNA–RBP Pair (2.5 MB ZIP)

Dataset S3 Significance Analysis of Microarray Results for Each Protein (11.8 MB ZIP)

Figure S1 Immunopurification Enrichment Profiles of Several RNA-Binding Proteins (A) Distribution of average Cy5/Cy3 fluorescence ratios from five independent microarray hybridizations analyzing Ssd1 targets. The enrichment distribution for mRNAs is shown in black, and the enrichment distribution for other annotated RNAs (i.e., nuclear introns, mitochondrion-encoded mRNAs, mitochondrial introns, snoRNAs, ribosomal RNAs, LSR1, NME1, SCR1, SRG1, and TLC1) is shown in red. The points correspond to an estimated distribution that was created by binning the average fluorescence ratios into 0.1 log_2_ unit bins from −7 to 7 log_2_ units. The lines correspond to a smoothed fit of the data [160]. We scaled the smoothed fit of the distribution to the binned data by making the maximum value of the smoothed fit data equal to the value in the bin with the largest number of RNAs.
(B) Same as in (A), except for Scp160. The results are the average of three independent microarray hybridizations.
(C) Same as in (A), except for Pab1. The results are the average of three independent microarray hybridizations.
(D) Same as in (A), except for Pub1. The results are the average of three independent microarray hybridizations.
(374 KB PDF)

Figure S2 Overrepresentation of Specific Classes of RNAs in Association with Specific RNA-Binding Proteins Enrichment of several classes of RNAs (rows) in target sets (1% FDR) of RBPs (columns). The significance of enrichment of the class of RNAs is represented as a heat map in which the color intensity corresponds to the negative log_10_ *p*-value, which was calculated using the hypergeometric density distribution function and corrected for multiple hypothesis testing using the Bonferroni method. RBPs whose targets are significantly enriched (*p* ≤ 0.05) for a specific class of RNAs are shown.
(219 KB PDF)

Figure S3 Specific Features of Post-Transcriptional Regulation May Be Linked to Broad-Specificity RNA-Binding Proteins Pearson correlations between IP enrichment with the RBP (columns) and selected characteristics of mRNAs (rows) are represented as a heat map. mRNAs that passed quality filtering for all nine RBPs were included in this analysis.
(231 KB PDF)

Table S1 Annotated and Putative RNA-Binding Proteins in Saccharomyces cerevisiae
(160 KB XLS)

Table S2 Summary of RNA-Binding Proteins in the Survey (49 KB XLS)

Table S3 Gene Ontology Terms Enriched in RNA-Binding Protein Target Sets (91 KB XLS)

Table S4 RNA Motifs Identified in RNA-Binding Protein Target Sequences (46 KB XLS)

Table S5 Description of Microarray Experiments and Groups Used for Group Normalization (41 KB XLS)

Text S1 Representation of RNA-Binding Proteins in This Study (24 KB DOC)

Text S2 Comments on the Immunopurification Method (51 KB DOC)

Text S3 Diverse RNA Enrichment Profiles among RNA-Binding Proteins (29 KB DOC)

Text S4 RNA-Binding Proteins That Preferentially Associate with RNAs Other Than Mature mRNAs Encoded by Nuclear Genes (68 KB DOC)

Text S5 Specific Features of Post-Transcriptional Regulation May Be Linked to Broad-Specificity RNA-Binding Proteins (38 KB DOC)

Text S6 Many RNA-Binding Proteins Appear to Bind Their Targets during Specific Stages in Their Lives (57 KB DOC)

Text S7 Putative RNA-Recognition Motifs (48 KB DOC)

Text S8 Many RNA-Binding Proteins Associated with Their Own Transcripts (32 KB DOC)

Text S9 Insights into the Functions of Specific RNA-Binding Proteins (49 KB DOC)

Text S10 Immunopurification Group Normalization (28 KB DOC)

Text S11 Significance Analysis of Microarrays (33 KB DOC)

## 

### Accession Numbers

Our microarray experiment data are publicly available from the Stanford Microarray Database and Gene Expression Omnibus.

## Abbreviations

ER: endoplasmic reticulum
FDR: false discovery rate
FIRE: finding informative regulatory elements
GO: gene ontology
IP: immunoaffinity purification
RBP: RNA-binding protein
REFINE: relative filtering by nucleotide enrichment
snoRNA: small nucleolar RNA
SRE: Smaug recognition element
TEV: tobacco etch virus
UTR: untranslated region
