# Supplementary figures and images for: Diverse RNA-Binding Proteins Interact with Functionally Related Sets of RNAs, Suggesting an Extensive Regulatory System
Source: PLoS Biol. 2008 Oct 28;6(10):e255. doi: 10.1371/journal.pbio.0060255 (PMC2573929; doi:10.1371/journal.pbio.0060255)

A

Ssd1

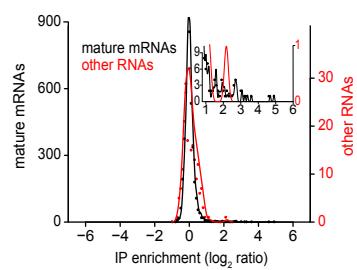

B

Scp160

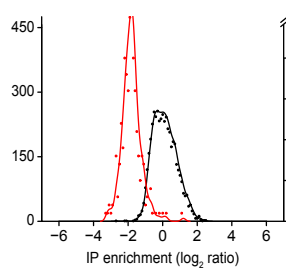

C

Pab1

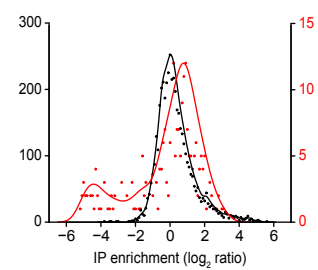

D

Pub1

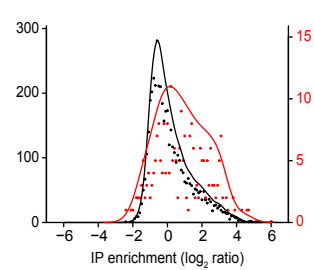

Supplement: Figure S1 — (A) Distribution of average Cy5/Cy3 fluorescence ratios from five independent microarray hybridizations analyzing Ssd1 targets. The enrichment distribution for mRNAs is shown in black, and the enrichment distribution for other annotated RNAs (i.e., nuclear introns, mitochondrion-encoded mRNAs, mitochondrial introns, snoRNAs, ribosomal RNAs, LSR1, NME1, SCR1, SRG1, and TLC1) is shown in red. The points correspond to an estimated distribution that was created by binning the average fluorescence ratios into 0.1 log2 unit bins from −7 to 7 log2 units. The lines correspond to a smoothed fit of the data [160]. We scaled the smoothed fit of the distribution to the binned data by making the maximum value of the smoothed fit data equal to the value in the bin with the largest number of RNAs. (B) Same as in (A), except for Scp160. The results are the average of three independent microarray hybridizations. (C) Same as in (A), except for Pab1. The results are the average of three independent microarray hybridizations. (D) Same as in (A), except for Pub1. The results are the average of three independent microarray hybridizations. (374 KB PDF) [file pbio.0060255.sg001.pdf]

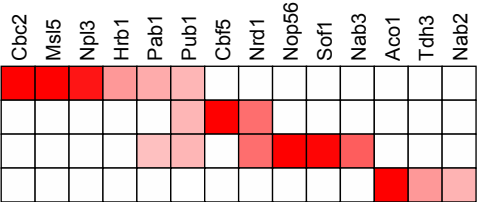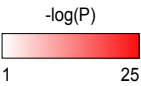

introns  
H/ACA snoRNAs  
C/D snoRNAs  
mitochondrion

Supplement: Figure S2 — Enrichment of several classes of RNAs (rows) in target sets (1% FDR) of RBPs (columns). The significance of enrichment of the class of RNAs is represented as a heat map in which the color intensity corresponds to the negative log10 p-value, which was calculated using the hypergeometric density distribution function and corrected for multiple hypothesis testing using the Bonferroni method. RBPs whose targets are significantly enriched (p ≤ 0.05) for a specific class of RNAs are shown. (219 KB PDF) [file pbio.0060255.sg002.pdf]

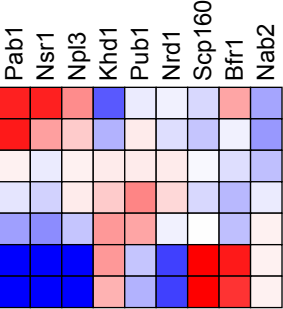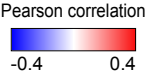

Supplement: Figure S3 — Pearson correlations between IP enrichment with the RBP (columns) and selected characteristics of mRNAs (rows) are represented as a heat map. mRNAs that passed quality filtering for all nine RBPs were included in this analysis. (231 KB PDF) [file pbio.0060255.sg003.pdf]
