# Supplementary material for: Diverse RNA-Binding Proteins Interact with Functionally Related Sets of RNAs, Suggesting an Extensive Regulatory System
Source: PLoS Biol. 2008 Oct 28;6(10):e255. doi: 10.1371/journal.pbio.0060255 (PMC2573929; doi:10.1371/journal.pbio.0060255)
Supplement: Text S1 — (24 KB DOC) [file pbio.0060255.sd004.doc]

**Representation of RNA-binding proteins in this study**

While this study is a “survey” of the RBP landscape, the 35 RBPs chosen represent a biased sampling of the predicted universe of ~560 RBPs. For instance, proteins containing canonical RNA recognition motifs (RRM) and K-homology (KH) RNA-binding domains are well-represented in this study (16/51 RRM and 5/8 KH), while several classes of proteins that were deemed less-likely to have specific mRNA binding activity are underrepresented, including components of the cytoplasmic ribosome (0/133), components of the nuclear spliceasome (2/59), tRNA-ligases (0/32) and mitochondrion-localized RBPs (0/101).
