# Supplementary material for: Diverse RNA-Binding Proteins Interact with Functionally Related Sets of RNAs, Suggesting an Extensive Regulatory System
Source: PLoS Biol. 2008 Oct 28;6(10):e255. doi: 10.1371/journal.pbio.0060255 (PMC2573929; doi:10.1371/journal.pbio.0060255)
Supplement: Text S2 — (51 KB DOC) [file pbio.0060255.sd005.doc]

**Comments on the immunopurification method**

We immunopurified 46 affinity-tagged proteins in *S. cerevisiae* and identified the associated RNAs by DNA microarray hybridization. Because the RBP-RNA interactions were not stabilized by cross-linking prior to lysis, there was potential for reassociation of the RNAs during the immunopurification procedure [1]. However, a recent study in *S. cerevisiae*, which employed a similar methodology, found no evidence for reassociation during RBP immunopurifications [2]. Moreover, in a previous study we found that levels of the mRNA targets of Puf3, identified by the same immunopurification procedure used in the present work, were specifically and consistently elevated in a Puf3 deletion mutant [3]. The physiological relevance of these interactions is further supported by subsequent analyses [4-9]. The striking functional themes among mRNAs associated with many of the RBPs investigated in this work, the strong links between the targets identified herein and genetic and functional results from previous work on several of these RBPs, and the phylogenetic conservation of RNA sequence motifs identified from the RBP target sets further support a physiological significance of many of the observed binding interactions.

We identified only a handful of associated mRNAs for two RBPs that have been shown to bind large sets of mRNAs (Mex67 and Upf1), suggesting some *bona fide* interactions were lost [10,11]. It is possible the dearth of targets we identified for Mex67 and Upf1 (and perhaps some other proteins) is at least partially due to immunopurification protocol variations. For instance, we observed a number of protocol variations that affect immunopurification results for some proteins, including divalent cation concentration (*e.g.* 1.8 mM MgCl_2_ vs 10 mM EDTA) in the immunopurification buffers and the beads used (*e.g.* porous vs nonporous beads).

**References**

1. Mili S, Steitz JA (2004) Evidence for reassociation of RNA-binding proteins after cell lysis: implications for the interpretation of immunoprecipitation analyses. Rna 10: 1692-1694.

2. Oeffinger M, Wei KE, Rogers R, DeGrasse JA, Chait BT, et al. (2007) Comprehensive analysis of diverse ribonucleoprotein complexes. Nat Methods 4: 951-956.

3. Gerber AP, Herschlag D, Brown PO (2004) Extensive association of functionally and cytotopically related mRNAs with Puf family RNA-binding proteins in yeast. PLoS Biol 2: E79.

4. Seay D, Hook B, Evans K, Wickens M (2006) A three-hybrid screen identifies mRNAs controlled by a regulatory protein. Rna 12: 1594-1600.

5. Prinz S, Aldridge C, Ramsey SA, Taylor RJ, Galitski T (2007) Control of signaling in a MAP-kinase pathway by an RNA-binding protein. PLoS ONE 2: e249.

6. Grigull J, Mnaimneh S, Pootoolal J, Robinson MD, Hughes TR (2004) Genome-wide analysis of mRNA stability using transcription inhibitors and microarrays reveals posttranscriptional control of ribosome biogenesis factors. Mol Cell Biol 24: 5534-5547.

7. Gasch AP, Moses AM, Chiang DY, Fraser HB, Berardini M, et al. (2004) Conservation and evolution of cis-regulatory systems in ascomycete fungi. PLoS Biol 2: e398.

8. Foat BC, Houshmandi SS, Olivas WM, Bussemaker HJ (2005) Profiling condition-specific, genome-wide regulation of mRNA stability in yeast. Proc Natl Acad Sci U S A 102: 17675-17680.

9. Elemento O, Slonim N, Tavazoie S (2007) A universal framework for regulatory element discovery across all genomes and data types. Mol Cell 28: 337-350.

10. Hieronymus H, Silver PA (2003) Genome-wide analysis of RNA-protein interactions illustrates specificity of the mRNA export machinery. Nat Genet 33: 155-161.

11. Johansson MJ, He F, Spatrick P, Li C, Jacobson A (2007) Association of yeast Upf1p with direct substrates of the NMD pathway. Proc Natl Acad Sci U S A 104: 20872-20877.
