# Supplementary material for: Diverse RNA-Binding Proteins Interact with Functionally Related Sets of RNAs, Suggesting an Extensive Regulatory System
Source: PLoS Biol. 2008 Oct 28;6(10):e255. doi: 10.1371/journal.pbio.0060255 (PMC2573929; doi:10.1371/journal.pbio.0060255)
Supplement: Text S3 — (29 KB DOC) [file pbio.0060255.sd006.doc]

**Diverse RNA enrichment profiles among RNA-binding proteins**

Thirty-seven of the 46 proteins associated with fewer than 500 RNAs each (1% FDR) (Figure 1A). As shown for Ssd1 in Figure s1A, many of these proteins have a tight distribution of enrichment factors, most RNAs showing no evidence for enrichment, with a “tail” of enrichment, similar to what we previously observed for the Puf proteins [1,2]. Nine proteins (Pab1, Pub1, Scp160, Npl3, Nrd1, Bfr1, Nab2, Khd1, Nsr1) associated with >500 RNAs each. These nine RBPs exhibited several different patterns of enrichment profiles (Figure S1B-D). The distribution of RNA enrichment factors was nearly bimodal for Pab1, Scp160, Npl3, Bfr1, Nab2 and Nsr1; for each of these proteins, mRNAs as a class were significantly enriched by association with the protein relative to many other types of RNAs (*e.g.* nuclear introns, mitochondrion-encoded mRNAs, snoRNAs, ribosomal RNAs) suggesting that these broadly specific RBPs selectively bind most or all mRNAs, but with varying efficiency. The distribution of mRNAs enriched by association with Scp160 was narrow (Figure S1B) – consistent with little differential specificity among mRNAs, while the distribution of Pab1-associated mRNAs (Figure S1C) had a long “tail” suggesting that differential binding of Pab1 to mRNAs could have significant functional consequences. RNAs other than nuclear-encoded mRNAs had a bimodal distribution of enrichment in Pab1 IPs (Figure S1C – red lines); RNAs encoded by the mitochondrial genome were significantly less enriched than other classes of RNAs. In contrast, Pub1 displayed a broad spectrum of enrichment (Figure S1D), but with no evidence for a bimodal distribution, nor were any groups of noncoding RNA significantly less enriched, as a group, than mRNAs.

**References**

1. Gerber AP, Herschlag D, Brown PO (2004) Extensive association of functionally and cytotopically related mRNAs with Puf family RNA-binding proteins in yeast. PLoS Biol 2: E79.

2. Shepard KA, Gerber AP, Jambhekar A, Takizawa PA, Brown PO, et al. (2003) Widespread cytoplasmic mRNA transport in yeast: identification of 22 bud-localized transcripts using DNA microarray analysis. Proc Natl Acad Sci U S A 100: 11429-11434.
