# Supplementary material for: Diverse RNA-Binding Proteins Interact with Functionally Related Sets of RNAs, Suggesting an Extensive Regulatory System
Source: PLoS Biol. 2008 Oct 28;6(10):e255. doi: 10.1371/journal.pbio.0060255 (PMC2573929; doi:10.1371/journal.pbio.0060255)
Supplement: Text S4 — (68 KB DOC) [file pbio.0060255.sd007.doc]

**RBPs that preferentially associate with RNAs other than mature mRNAs encoded by nuclear genes**

Fourteen of the proteins we surveyed specifically associated with RNAs other than mature mRNAs encoded by nuclear genes (Figure S2). Their specific targets included intron-containing transcripts (Cbc2, Msl5, Npl3, Hrb1, Pab1, Pub1), H/ACA box snoRNAs (Cbf5, Nrd1, Pub1), C/D box snoRNAs (Nop56, Sof1, Nab3, Nrd1, Pub1, Pab1) and mitochondrial mRNAs (Aco1, Tdh3, Nab2). Several of these proteins have previously been shown to be associated with specific classes of RNA (Cbc2, Msl5, Npl3, Cbf5, Nrd1, Nop56, Sof1, Nab3), and therefore provide *de facto* positive controls (Table S2). For example, intron-containing (presumably intranuclear) transcripts were preferentially enriched by binding to Msl5, the splicing-branchpoint binding protein (P < 10^-91^) [1], and by Cbc2, a component of nuclear cap binding complex and splicing commitment complex, (P < 10^-133^) [2]. The protein that catalyzes pseudouridylation via guide H/ACA box snoRNAs, Cbf5 [3], bound H/ACA box snoRNAs (23/23 most enriched RNAs P < 10^-80^), while Nop56 (also called Sik1), a C/D box snoRNP component, specifically bound to C/D box snoRNAs (P < 10^-39^) [3,4]. Nrd1, a protein involved in termination of non-polyadenylated RNA polymerase II transcripts [5-8], was specifically associated with both types of snoRNAs (P < 10^-8^ and 10^-8^ for H/ACA box snoRNAs and C/D box snoRNAs, respectively) and with many newly discovered “intergenic” transcripts (*e.g.* SRG1: FDR=0, rank = 5) [9]. Npl3, a predominantly nuclear protein involved in mRNA processing and export and functionally linked to the splicing machinery [10,11], preferentially associated with intron-containing transcripts (P < 10^-21^).

In several instances the association with specific class of RNAs that were identified is unprecedented. For example, Aco1, a TCA cycle enzyme [12], which has recently been implicated in maintaining mitochondrial genome integrity [13], selectively binds mRNAs encoded by the mitochondrial genome (P < 10^-38^). Nab2, a predominantly nuclear protein involved in mRNA processing [14,15], also selectively associated with RNAs encoded by the mitochondrial genome (P = 0.007) in addition to hundreds of mRNAs encoded by nuclear genes. Pub1, a predominantly cytoplasmic protein implicated in regulating the stability of hundreds of mRNAs [16], selectively associated with nuclear introns (P = 0.01) and both types of snoRNAs (P = 0.01 for both H/ACA box snoRNAs and C/D box snoRNAs) as well as hundreds of mRNAs encoded by nuclear genes.

**References**

1. Rutz B, Seraphin B (2000) A dual role for BBP/ScSF1 in nuclear pre-mRNA retention and splicing. Embo J 19: 1873-1886.

2. Colot HV, Stutz F, Rosbash M (1996) The yeast splicing factor Mud13p is a commitment complex component and corresponds to CBP20, the small subunit of the nuclear cap-binding complex. Genes Dev 10: 1699-1708.

3. Watkins NJ, Gottschalk A, Neubauer G, Kastner B, Fabrizio P, et al. (1998) Cbf5p, a potential pseudouridine synthase, and Nhp2p, a putative RNA-binding protein, are present together with Gar1p in all H BOX/ACA-motif snoRNPs and constitute a common bipartite structure. Rna 4: 1549-1568.

4. Lafontaine DL, Tollervey D (2000) Synthesis and assembly of the box C+D small nucleolar RNPs. Mol Cell Biol 20: 2650-2659.

5. Arigo JT, Eyler DE, Carroll KL, Corden JL (2006) Termination of cryptic unstable transcripts is directed by yeast RNA-binding proteins Nrd1 and Nab3. Mol Cell 23: 841-851.

6. Steinmetz EJ, Conrad NK, Brow DA, Corden JL (2001) RNA-binding protein Nrd1 directs poly(A)-independent 3'-end formation of RNA polymerase II transcripts. Nature 413: 327-331.

7. Thiebaut M, Kisseleva-Romanova E, Rougemaille M, Boulay J, Libri D (2006) Transcription termination and nuclear degradation of cryptic unstable transcripts: a role for the nrd1-nab3 pathway in genome surveillance. Mol Cell 23: 853-864.

8. Vasiljeva L, Buratowski S (2006) Nrd1 interacts with the nuclear exosome for 3' processing of RNA polymerase II transcripts. Mol Cell 21: 239-248.

9. Martens JA, Laprade L, Winston F (2004) Intergenic transcription is required to repress the Saccharomyces cerevisiae SER3 gene. Nature 429: 571-574.

10. Flach J, Bossie M, Vogel J, Corbett A, Jinks T, et al. (1994) A yeast RNA-binding protein shuttles between the nucleus and the cytoplasm. Mol Cell Biol 14: 8399-8407.

11. Lee MS, Henry M, Silver PA (1996) A protein that shuttles between the nucleus and the cytoplasm is an important mediator of RNA export. Genes Dev 10: 1233-1246.

12. Gangloff SP, Marguet D, Lauquin GJ (1990) Molecular cloning of the yeast mitochondrial aconitase gene (ACO1) and evidence of a synergistic regulation of expression by glucose plus glutamate. Mol Cell Biol 10: 3551-3561.

13. Chen XJ, Wang X, Kaufman BA, Butow RA (2005) Aconitase couples metabolic regulation to mitochondrial DNA maintenance. Science 307: 714-717.

14. Anderson JT, Wilson SM, Datar KV, Swanson MS (1993) NAB2: a yeast nuclear polyadenylated RNA-binding protein essential for cell viability. Mol Cell Biol 13: 2730-2741.

15. Hector RE, Nykamp KR, Dheur S, Anderson JT, Non PJ, et al. (2002) Dual requirement for yeast hnRNP Nab2p in mRNA poly(A) tail length control and nuclear export. Embo J 21: 1800-1810.

16. Duttagupta R, Tian B, Wilusz CJ, Khounh DT, Soteropoulos P, et al. (2005) Global analysis of Pub1p targets reveals a coordinate control of gene expression through modulation of binding and stability. Mol Cell Biol 25: 5499-5513.
