# Supplementary material for: Diverse RNA-Binding Proteins Interact with Functionally Related Sets of RNAs, Suggesting an Extensive Regulatory System
Source: PLoS Biol. 2008 Oct 28;6(10):e255. doi: 10.1371/journal.pbio.0060255 (PMC2573929; doi:10.1371/journal.pbio.0060255)
Supplement: Text S5 — (38 KB DOC) [file pbio.0060255.sd008.doc]

Specific features of post-transcriptional regulation may be linked to broad-specificity RNA-binding proteins

Immunoaffinity enrichment of mRNAs associated with Pab1, Nsr1, and Npl3 correlated with ribosome occupancy (Pearson correlation = 0.35, 0.35 and 0.18, respectively) (Figure S3). The Pab1 link is described in the main text.

In contrast, immunoaffinity enrichment with Khd1 negatively correlated with ribosome occupancy (r = -0.26). This example is described in the main text.

Association of mRNAs with Pub1 correlated with both 3’-UTR and 5’-UTR length (r = 0.19 and 0.14, respectively). Pub1 is a predominantly cytoplasmic protein that binds with high affinity to U-rich stretches in the 5’-UTR and 3’-UTR of mRNAs [1-4]. Because these sequences are common and found in virtually all mRNAs, it is possible that Pub1 enrichment reflects not only the affinity and accessibility of these sites to Pub1, but also the number of U-rich stretches, and thus the number of Pub1 proteins bound per mRNA. Both the number and density of poly(U) stretches of length seven is significantly higher in the untranslated regions of Pub1 IP targets (1% FDR) compared to nontargets (average number in UTRs of targets = 0.83, nontargets = 0.19, P < 10^-15^; number per 100 nucleotides in UTRs of targets = 0.29, nontargets = 0.08, P < 10^-15^). A disproportionate fraction of the 1421 mRNAs associated with Pub1 at 1% FDR encode proteins localized to membranes (396, P < 10^-20^), including the endoplasmic reticulum (164, P < 10^-14^), plasma membrane (106, P < 10^-9^), and vacuole (94, P < 10^-8^). mRNAs encoding cell wall proteins (41, P < 10^-4^) and proteins that function in the electron transport chain (19, P < 10^-4^) were also overrepresented in the Pub1 targets . Pub1-associated mRNAs identified in our study significantly overlap a previously reported set of Pub1 targets identified by an immunopurification method [2].

**References**

1. Anderson JT, Paddy MR, Swanson MS (1993) PUB1 is a major nuclear and cytoplasmic polyadenylated RNA-binding protein in Saccharomyces cerevisiae. Mol Cell Biol 13: 6102-6113.

2. Duttagupta R, Tian B, Wilusz CJ, Khounh DT, Soteropoulos P, et al. (2005) Global analysis of Pub1p targets reveals a coordinate control of gene expression through modulation of binding and stability. Mol Cell Biol 25: 5499-5513.

3. Ruiz-Echevarria MJ, Peltz SW (2000) The RNA binding protein Pub1 modulates the stability of transcripts containing upstream open reading frames. Cell 101: 741-751.

4. Vasudevan S, Peltz SW (2001) Regulated ARE-mediated mRNA decay in Saccharomyces cerevisiae. Mol Cell 7: 1191-1200.
