# Supplementary material for: Diverse RNA-Binding Proteins Interact with Functionally Related Sets of RNAs, Suggesting an Extensive Regulatory System
Source: PLoS Biol. 2008 Oct 28;6(10):e255. doi: 10.1371/journal.pbio.0060255 (PMC2573929; doi:10.1371/journal.pbio.0060255)
Supplement: Text S6 — (57 KB DOC) [file pbio.0060255.sd009.doc]

**Many RNA-binding proteins appear to bind their targets during specific stages in their lives**

Three proteins (Msl5, Nrd1, and Pub1) bound preferentially to intron-containing transcripts compared to all RNAs (25% mean enrichment of intron-containing transcripts over median IP enrichment of all RNAs) (Figure 3). Msl5 and Nrd1 are predominantly localized to the nucleus and have been shown to interact with intron-containing transcripts [1,2] and transcribing RNA polymerase II [3,4], respectively. In contrast, Pub1 is predominantly cytoplasmic [5] and has not previously been linked to mRNA processing of intron-containing transcripts. In support of Pub1’s association with pre-mRNAs, a small fraction of Pub1 molecules are localized to the nucleus [5,6] and Pub1 has been shown to physically interact with several predominantly nuclear RBPs, including Nrd1, Nab3, Npl3, and Nab2 [5-7].

Three proteins (Cbc2, Npl3, and Pab1) were preferentially associated with both intron-containing transcripts and mature mRNAs derived from intron-containing transcripts (Figure 3, see main text).

One protein, Nsr1, preferentially associated with RNAs containing exon sequences from spliced transcripts (mean enrichment of exonic sequences = 1.4), but not the corresponding intron sequences (mean enrichment of intronic sequences = 0.7) (Figure 3). This was surprising because Nsr1 is predominantly localized to the nucleolus, where it is required for rRNA processing [8]. Indeed, several ribosomal precursor RNAs were among the RNAs most enriched in Nsr1 affinity purifications. This result suggests that in addition to its nucleolar role in ribosome biogenesis, Nsr1 may have a role in regulating specific mRNAs in the cytoplasm. The 527 mRNAs associated with Nsr1 at 1% FDR included a disproportionate fraction of mRNAs encoding components of the cytosolic ribosome (50, P < 10^-11^) and the mitochondrial ribosome (27, P < 10^-5^). Among mature mRNAs that were derived from intron-containing transcripts for which we obtained high-quality measurements, Nsr1 preferentially associated with those that encode components of the cytosolic ribosome (50% ribosomal proteins versus 20% non-ribosomal proteins at 1% FDR, P < 10^-11^, one-sided binomial test).

In contrast, intron-containing transcripts were underrepresented in the RNAs associated with Scp160 and Bfr1 (mean enrichment of intronic sequences = 0.4 and 0.2, respectively) (Figure 3). This result is consistent with previous studies indicating that these proteins bind mRNAs during translation in the cytoplasm [9,10].

**References**

1. Abovich N, Rosbash M (1997) Cross-intron bridging interactions in the yeast commitment complex are conserved in mammals. Cell 89: 403-412.

2. Rutz B, Seraphin B (2000) A dual role for BBP/ScSF1 in nuclear pre-mRNA retention and splicing. Embo J 19: 1873-1886.

3. Steinmetz EJ, Brow DA (1998) Control of pre-mRNA accumulation by the essential yeast protein Nrd1 requires high-affinity transcript binding and a domain implicated in RNA polymerase II association. Proc Natl Acad Sci U S A 95: 6699-6704.

4. Conrad NK, Wilson SM, Steinmetz EJ, Patturajan M, Brow DA, et al. (2000) A yeast heterogeneous nuclear ribonucleoprotein complex associated with RNA polymerase II. Genetics 154: 557-571.

5. Anderson JT, Paddy MR, Swanson MS (1993) PUB1 is a major nuclear and cytoplasmic polyadenylated RNA-binding protein in Saccharomyces cerevisiae. Mol Cell Biol 13: 6102-6113.

6. Apponi LH, Kelly SM, Harreman MT, Lehner AN, Corbett AH, et al. (2007) An interaction between two RNA binding proteins, Nab2 and Pub1, links mRNA processing/export and mRNA stability. Mol Cell Biol 27: 6569-6579.

7. Collins SR, Kemmeren P, Zhao XC, Greenblatt JF, Spencer F, et al. (2007) Toward a comprehensive atlas of the physical interactome of Saccharomyces cerevisiae. Mol Cell Proteomics 6: 439-450.

8. Lee WC, Zabetakis D, Melese T (1992) NSR1 is required for pre-rRNA processing and for the proper maintenance of steady-state levels of ribosomal subunits. Mol Cell Biol 12: 3865-3871.

9. Lang BD, Fridovich-Keil JL (2000) Scp160p, a multiple KH-domain protein, is a component of mRNP complexes in yeast. Nucleic Acids Res 28: 1576-1584.

10. Lang BD, Li A, Black-Brewster HD, Fridovich-Keil JL (2001) The brefeldin A resistance protein Bfr1p is a component of polyribosome-associated mRNP complexes in yeast. Nucleic Acids Res 29: 2567-2574.
