# Supplementary material for: Diverse RNA-Binding Proteins Interact with Functionally Related Sets of RNAs, Suggesting an Extensive Regulatory System
Source: PLoS Biol. 2008 Oct 28;6(10):e255. doi: 10.1371/journal.pbio.0060255 (PMC2573929; doi:10.1371/journal.pbio.0060255)
Supplement: Text S7 — (48 KB DOC) [file pbio.0060255.sd010.doc]

**Putative RNA-recognition motifs**

The motifs we identified for Puf3, Puf4, Puf5, Pub1, Nab2, Nrd1 and Nab3 match previously described binding sites for the corresponding RBPs, validating our approach and suggesting that many of the RBP-RNA interactions we measured are likely to be directly mediated by these elements (Figure 5 and Table S4). The three most significant predicted motifs identified in our analysis correspond to the known recognition elements for Puf3, Puf4, and Puf5 [1]. The Puf3-1, Puf4-1, and Puf5-1 motifs are all extremely enriched and preferentially conserved in the 3’-UTRs of their RBP targets, and also show significant enrichment in other regions of target sequences. The next motif, Pub1-1, is enriched in Pub1 target 3’-UTRs and is similar to U-rich RNA elements which have been previously reported as binding sites for Pub1 [2-5]. The Nab2-1 element we identified matches a motif with consensus AAAAAG previously identified by bioinformatic analysis of Nab2 target RNAs [6] and is in agreement with the reported high affinity (~10 nM) binding of Nab2 to 25-mer poly(A) RNA *in vitro* [7]. The Nrd1 and Nab3 motifs are described in the main text.

The two most significant novel motifs, Puf2-1 (UAAUAAUUW) and Ssd1-1 (AKUCAUUCCUU), are described in the main text. The Pab1-1 motif contains a UA-repeat consensus which differs from the canonical poly(A) binding preference for Pab1, and this motif is also significantly enriched in sets of targets for multiple distinct RBPs (Table S4). It is therefore unlikely that the Pab1-1 element is involved in specifying direct interactions with Pab1, but the reason for its significant overrepresentation in targets of Pab1 and other RBPs remains unclear. The Khd1-1 motif is not found in the E1 element (bases 622-900) of the ASH1 mRNA coding sequence that has previously been identified as directly binding to Khd1 [8], and may therefore represent a binding site for an unknown factor that regulates a set of mRNAs that overlaps extensively with the targets of Khd1. Two other novel motifs include Nsr1-1, which is significantly enriched in 5’-UTRs of target mRNAs, and Pin4-1, which is selectively enriched in the 3’-UTRs of target mRNAs (Figure 5). YLL032C-1 is another novel motif, which is notably enriched in both the 3’- and 5’-UTRs as well as coding sequences of targets (Figure 5).

**Limitations in our bioinformatic motif-finding analyses**

One feature apparent from our motif analysis is the propensity for individual motifs to display significant enrichment in targets of multiple RBPs, often mirroring the significant degree of overlap we observed among RNA targets bound by distinct RBPs (Table S4, Figure 1B and Figure 4A). In some cases a motif enriched in multiple RBP target sets may be involved in directly specifying multiple interactions – indeed we’ve interpreted the Puf2-1 element as interacting with both of the evolutionarily-related Puf2 and Puf1 proteins, and the Nrd1-1 motif matches the reported binding sites of the individual Nrd1 and Nab3 proteins, which physically interact in a complex. In other cases enrichment of motif sequences in multiple target sets is more plausibly attributable to functionally overlapping regulatory systems. The large degree of motif co-enrichment observed in our analysis is consistent with combinatorial regulation by a highly interconnected regulatory network. However, the prevalence of motif enrichment due to functionally overlapping regulatory networks also makes it more challenging to accurately identify which elements are directly responsible for mediating specific interactions. This difficulty represents an important limitation of computational regulatory element identification and underscores the critical importance of direct biochemical and genetic assays to confirm and characterize the roles of sequence elements that have been predicted computationally.

For at least eight RBPs (Cbf5, Hrb1, Msl5, Nab6, Npl3, Sgn1, Ski2, Tdh3) our systematic analysis did not identify any sequence motifs with statistically significant enrichment. It is possible that we failed to identify motifs for some of these RBPs because the deliberately stringent statistical criteria we applied were overly strict. For Msl5, the branchpoint binding protein involved in intron splicing, a more detailed inspection revealed that this was in fact the case. The 3’-UTRs of Msl5 targets annotated as lacking introns were significantly more likely to contain exact matches to the strict branchpoint consensus sequence ‘UACUAAC’ (15% targets versus 1% non-targets, P < 10^-6^), suggesting that either these transcripts contain novel introns or that Msl5 may play a splicing-independent role in the regulation of these RNAs. Although both REFINE and FIRE identified motifs with consensus sequences similar to the branchpoint signal, neither passed the stringent significance thresholds we imposed (Table S4). Another possibility is that RBPs for which we failed to predict sequence motifs might recognize RNA structural elements or features primarily present in coding sequences (see Vts1 description in main text).

**References**

1. Gerber AP, Herschlag D, Brown PO (2004) Extensive association of functionally and cytotopically related mRNAs with Puf family RNA-binding proteins in yeast. PLoS Biol 2: E79.

2. Vasudevan S, Peltz SW (2001) Regulated ARE-mediated mRNA decay in Saccharomyces cerevisiae. Mol Cell 7: 1191-1200.

3. Matunis MJ, Matunis EL, Dreyfuss G (1993) PUB1: a major yeast poly(A)+ RNA-binding protein. Mol Cell Biol 13: 6114-6123.

4. Duttagupta R, Tian B, Wilusz CJ, Khounh DT, Soteropoulos P, et al. (2005) Global analysis of Pub1p targets reveals a coordinate control of gene expression through modulation of binding and stability. Mol Cell Biol 25: 5499-5513.

5. Anderson JT, Paddy MR, Swanson MS (1993) PUB1 is a major nuclear and cytoplasmic polyadenylated RNA-binding protein in Saccharomyces cerevisiae. Mol Cell Biol 13: 6102-6113.

6. Kim Guisbert K, Duncan K, Li H, Guthrie C (2005) Functional specificity of shuttling hnRNPs revealed by genome-wide analysis of their RNA binding profiles. Rna 11: 383-393.

7. Hector RE, Nykamp KR, Dheur S, Anderson JT, Non PJ, et al. (2002) Dual requirement for yeast hnRNP Nab2p in mRNA poly(A) tail length control and nuclear export. Embo J 21: 1800-1810.

8. Paquin N, Menade M, Poirier G, Donato D, Drouet E, et al. (2007) Local activation of yeast ASH1 mRNA translation through phosphorylation of Khd1p by the casein kinase Yck1p. Mol Cell 26: 795-809.
