# Supplementary material for: Diverse RNA-Binding Proteins Interact with Functionally Related Sets of RNAs, Suggesting an Extensive Regulatory System
Source: PLoS Biol. 2008 Oct 28;6(10):e255. doi: 10.1371/journal.pbio.0060255 (PMC2573929; doi:10.1371/journal.pbio.0060255)
Supplement: Text S8 — (32 KB DOC) [file pbio.0060255.sd011.doc]

**Many RNA-binding proteins associated with their own transcripts**

Autoregulation is common among regulatory proteins [1]. There are many published examples of RBPs that bind their own mRNAs, including two proteins in the survey (Nrd1 and Nab2), which negatively autoregulate their respective transcripts [2,3]. We found NRD1 and NAB2 mRNAs significantly enriched in IPs with their respective RBPs (0% FDR and 1.1% FDR, respectively). In total, at least 17 of 46 proteins in the survey (Aco1, Atg8, Hrb1, Khd1, Msl5, Npl3, Nrd1, Pin4, Puf1, Puf2, Puf4, Rna15, Scp160, Ski2, Vts1, Yll032c, Ypl184c) appeared to bind their own transcript (1% FDR). Several lines of evidence suggest, however, that at least some of these interactions are indirect. We identified RNA targets of seven RBPs in the presence and absence of Mg^2+^ (Bfr1, Dhh1, Gbp2, Khd1, Nab2, Scp160, Ssd1 – Dataset S1), including five RBPs (Gbp2, Khd1, Nab2, Scp160, Ssd1) that associated with their own transcript in the presence of Mg^2+^. In general, the identified targets were similar under these two conditions, but for one RBP the association with its own mRNA was selectively lost in the absence of Mg^2+^ (Ssd1). Of the seven RBPs that appeared to bind their own transcripts and for which we or others have identified a putative recognition element (Pin4, Puf1, Puf2, Puf4, Ssd1, Vts1, Msl5) (Figure 5), only one of the respective mRNAs (PUF2) clearly contains the corresponding element (Figure 6G). Further, the TAP-tagged mRNAs used in our study do not contain their endogenous 3’-UTRs, where many RBP-mRNA interactions occur. We therefore suspect that some of these interactions are indirect, perhaps because our immunoaffinity procedure can, in principle, isolate nascent TAP-tagged polypeptides while they are still associated with the ribosome and cognate mRNA. Further experiments are required to address the extent, roles and mechanisms of autoregulation by these RBPs.

**References**

1. Alon U (2007) Network motifs: theory and experimental approaches. Nat Rev Genet 8: 450-461.

2. Steinmetz EJ, Conrad NK, Brow DA, Corden JL (2001) RNA-binding protein Nrd1 directs poly(A)-independent 3'-end formation of RNA polymerase II transcripts. Nature 413: 327-331.

3. Roth KM, Wolf MK, Rossi M, Butler JS (2005) The nuclear exosome contributes to autogenous control of NAB2 mRNA levels. Mol Cell Biol 25: 1577-1585.
