# Supplementary material for: Diverse RNA-Binding Proteins Interact with Functionally Related Sets of RNAs, Suggesting an Extensive Regulatory System
Source: PLoS Biol. 2008 Oct 28;6(10):e255. doi: 10.1371/journal.pbio.0060255 (PMC2573929; doi:10.1371/journal.pbio.0060255)
Supplement: Text S9 — (49 KB DOC) [file pbio.0060255.sd012.doc]

**Insights into the functions of specific RNA-binding proteins**

Khd1 was first characterized in a screen for putative RBPs that affect localization of ASH1 mRNA to the bud-tip and repress its translation in the mother cell [1]. Khd1 contains three KH RNA-binding domains and is predominantly cytoplasmic. ASH1 is the only previously identified RNA target of Khd1.

We found that Khd1 was associated with 591 annotated mRNAs at 1% local FDR. Among its targets, mRNAs encoding proteins localized to the “cell periphery”, including cell wall (47, P < 10^-19^), plasma membrane (49, P < 10^-4^), bud (33, P < 10^-5^), site of polarized growth (36, P <10^-4^), and cortical cytoskeletal patch (13, P = 0.002) were significantly enriched. Nine of the 22 mRNAs that have been shown to localize to the bud-tip via association with the She2-She3-Myo4 complex, including ASH1, were also targets of Khd1 [2]. Although mRNAs encoding cell periphery proteins were highly over-represented among Khd1 targets, they comprised only a minority of the 591 Khd1 targets. Khd1 also preferentially associated with mRNAs encoding regulatory proteins, including transcription factors (41, P = 0.002), signaling proteins (39, P = 0.003), and RBPs (20), including 13 of the 40 proteins in this survey and four Puf proteins. Across all mRNAs, immunoaffinity enrichment with Khd1 was negatively correlated with ribosome occupancy (r = -0.26) (Figure S3). These data suggest that Khd1 represses translation initiation of hundreds to thousands of mRNAs, perhaps during their transport to specific cellular loci.

A further analysis of Khd1, which used these data to design experiments to map Khd1 binding sites in several target mRNAs, characterize a putative Khd1 RNA-recognition element and test some of the functional consequences of Khd1-mRNA interactions was recently published [3].

Gbp2 contains three RRMs and a serine-arginine rich (SR) motif and associates with the transcription/export (TREX) complex [4]. Gpb2 associates with actively transcribed genes [4] and is believed to be involved in the export of at least some of these mRNAs from the nucleus to the cytoplasm [5]. Gbp2 was also shown to associated with polysomes [5]. Gbp2 overexpression leads to reduced growth rate relative to wild-type cells [5].

We found 234 annotated mRNAs associated with Gbp2. 134 of these mRNAs encode proteins localized to the nucleus (P < 10^-17^). Many of these proteins are further localized to the nucleolus (39, P < 10^-10^) and are involved in ribosome biogenesis (44, P < 10^-10^), while another large subset are localized to the nucleoplasm and chromosomes (50, P < 10^-5^) and are involved in chromosome organization and transcription (57, P < 10^-6^). Many of these mRNAs encode proteins that function together in more specialized processes, such as 35S, 27S and 20S RNA processing, chromatin modification, telomere organization, and as part of the RNA polymerase II general transcription factor complex.

Given the available data on Gbp2, we speculate that Gbp2 binds targets co-transcriptionally and remains bound during nuclear export and perhaps translation. Gbp2 is actively recruited back into the nucleus by site-specific methylation [5], and perhaps Gbp2 facilitates co-translational nuclear import of the nascent proteins encoded by mRNA targets.

We were unable to identify a putative RNA-recognition element for Gbp2; its reported association with multiple sites along transcripts [4] suggests Gbp2 may recognize a highly degenerate RNA sequence or structure, which is difficult to identify bioinformatically with the available data.

**References**

1. Irie K, Tadauchi T, Takizawa PA, Vale RD, Matsumoto K, et al. (2002) The Khd1 protein, which has three KH RNA-binding motifs, is required for proper localization of ASH1 mRNA in yeast. Embo J 21: 1158-1167.

2. Shepard KA, Gerber AP, Jambhekar A, Takizawa PA, Brown PO, et al. (2003) Widespread cytoplasmic mRNA transport in yeast: identification of 22 bud-localized transcripts using DNA microarray analysis. Proc Natl Acad Sci U S A 100: 11429-11434.

3. Hasegawa Y, Irie K, Gerber AP (2008) Distinct roles for Khd1p in the localization and expression of bud-localized mRNAs in yeast. pp. rna.1016508.

4. Hurt E, Luo MJ, Rother S, Reed R, Strasser K (2004) Cotranscriptional recruitment of the serine-arginine-rich (SR)-like proteins Gbp2 and Hrb1 to nascent mRNA via the TREX complex. Proc Natl Acad Sci U S A 101: 1858-1862.

5. Windgassen M, Krebber H (2003) Identification of Gbp2 as a novel poly(A)+ RNA-binding protein involved in the cytoplasmic delivery of messenger RNAs in yeast. EMBO Rep 4: 278-283.
