# Supplementary material for: Diverse RNA-Binding Proteins Interact with Functionally Related Sets of RNAs, Suggesting an Extensive Regulatory System
Source: PLoS Biol. 2008 Oct 28;6(10):e255. doi: 10.1371/journal.pbio.0060255 (PMC2573929; doi:10.1371/journal.pbio.0060255)
Supplement: Text S10 — (28 KB DOC) [file pbio.0060255.sd013.doc]

## Immunopurification group normalization

1. Select experiment groups. Fifteen groups (three cDNA microarray and twelve oligonucleotide microarray groups) were selected based on date of immunopurification, buffer composition, array platform, and clustering of array experiments (Table S5)*.*
2. Select high quality spots for each array. For groups 1,3-7,9-15 spots were filtered for regression correlation >0.6 and channel 1 or channel 2 median intensity/median background intensity >2.5. For groups 2 and 8, spots were filtered for regression correlation >0.6 or channel 1 or channel 2 median intensity/median background intensity >2.5.
3. Select features that consistently had high-quality measurements across the group. Only features that passed filtering in at least 60% of experiments were used. While this potentially eliminates low-abundance RNAs bound by a specific RBP, it is helpful to have many measurements for each feature to estimate the true null value.
4. Center the median log_2_ value of each array at 0.
5. Calculate the median value for each feature across the group. Bfr1, Nab2, Npl3, Nsr1, Pab1, Pub1 and Scp160 experiments were not included in median calculations because they have low overall correlation with mock IPs (and most other protein IPs). We used the median value instead of the mean because it is more conservative and is likely a more accurate estimate of the null value.
6. Subtract median value from each experiment.
7. Merge median centered data from all groups from same array platform (Dataset S1). Features that measured Ty transposable elements were removed prior to subsequent analyses. For oligonucleotide microarrays, features that measured a single mRNA at multiple locations (* _TIL_*) along the mRNA were removed prior to subsequent analyses.
