# Supplementary material for: Diverse RNA-Binding Proteins Interact with Functionally Related Sets of RNAs, Suggesting an Extensive Regulatory System
Source: PLoS Biol. 2008 Oct 28;6(10):e255. doi: 10.1371/journal.pbio.0060255 (PMC2573929; doi:10.1371/journal.pbio.0060255)
Supplement: Text S11 — (33 KB DOC) [file pbio.0060255.sd014.doc]

**Significance analysis of microarrays**

I Oligonucleotide arrays groups 1-12

1. Remove features with missing values in >4 of 13 mock experiments.
2. For proteins with two replicates, remove features with missing values in either experiment. For proteins with 3-5 replicates, remove features with missing values in >2 experiments.
3. Run SAM for each protein against thirteen Mg^2+^ mock IPs.

II cDNA arrays group 13

1. Remove features with missing values in >1 of 4 mock experiments.
2. Remove features with missing values in >1 of 3-4 protein experiments.
3. Run SAM for each protein against four mock IP arrays as described above.

III cDNA arrays groups 14 and 15

1. Remove features with missing values in either protein experiment.
2. Remove features with missing values in >4 of 10 other protein experiments.
3. Run SAM for each protein against other 10 protein IP arrays.

In most cases IP targets were classified as RNAs with an FDR (q-value) <1. For Ssd1, Khd1 and Puf1-5, targets were classified as RNAs with a local FDR <1, which is a more stringent threshold [1]. A more stringent threshold for these experiments was used because the functional coherency among putative targets and the fraction of putative targets that contained RNA-recognition elements dropped dramatically above the 1% local FDR threshold. For Ssd1 and Puf1-5, there was an infliction in the number of targets called significant near the 1% local FDR threshold, which supports the use of this more stringent threshold. For She2, targets defined in Sheperd *et al.* were used [2].

**References**

1. Efron B, Tibshirani R (2002) Empirical bayes methods and false discovery rates for microarrays. Genet Epidemiol 23: 70-86.

2. Shepard KA, Gerber AP, Jambhekar A, Takizawa PA, Brown PO, et al. (2003) Widespread cytoplasmic mRNA transport in yeast: identification of 22 bud-localized transcripts using DNA microarray analysis. Proc Natl Acad Sci U S A 100: 11429-11434.
